# *TGM2*, *HMGA2*, *FXYD3*, and *LGALS4* genes as biomarkers in acquired oxaliplatin resistance of human colorectal cancer: A systems biology approach

**DOI:** 10.1371/journal.pone.0289535

**Published:** 2023-08-03

**Authors:** Tayebeh Cheraghi-shavi, Razieh Jalal, Zarrin Minuchehr

**Affiliations:** 1 Faculty of Science, Department of Chemistry, Ferdowsi University of Mashhad, Mashhad, Iran; 2 Institute of Biotechnology, Novel Diagnostics and Therapeutics Research Group, Ferdowsi University of Mashhad, Mashhad, Iran; 3 Systems Biotechnology Department, National Institute of Genetic Engineering and Biotechnology, Tehran, Iran; Monash University Malaysia, MALAYSIA

## Abstract

Acquired resistance to oxaliplatin is considered as the primary reason for failure in colorectal cancer (CRC) therapy. Identifying the underlying resistance mechanisms may improve CRC treatment. The present study aims to identify the key genes involved in acquired oxaliplatin-resistant in CRC by confirming the oxaliplatin resistance index (OX-RI). To this aim, two public microarray datasets regarding oxaliplatin-resistant CRC cells with different OX-RI, GSE42387, and GSE76092 were downloaded from GEO database to identify differentially expressed genes (DEGs). The results indicated that the OX-RI affects the gene expression pattern significantly. Then, 54 common DEGs in both datasets including 18 up- and 36 down-regulated genes were identified. Protein-protein interaction (PPI) analysis revealed 13 up- (*MAGEA6*, *TGM2*, *MAGEA4*, *SCHIP1*, *ECI2*, *CD33*, *AKAP12*, *MAGEA12*, *CALD1*, *WFDC2*, *VSNL1*, *HMGA2*, and *MAGEA2B*) and 12 down-regulated (*PDZK1IP1*, *FXYD3*, *ALDH2*, *CEACAM6*, *QPRT*, *GRB10*, *TM4SF4*, *LGALS4*, *ALDH3A1*, *USH1C*, *KCNE3*, and *CA12*) hub genes. In the next step, two novel up-regulated hub genes including *ECI2* and *SCHIP1* were identified to be related to oxaliplatin resistance. Functional enrichment and pathway analysis indicated that metabolic pathways, proliferation, and epithelial-mesenchymal transition may play dominant roles in CRC progression and oxaliplatin resistance. In the next procedure, two in vitro oxaliplatin-resistant sub-lines including HCT116/OX-R4.3 and HCT116/OX-R10 cells with OX-IR 3.93 and 10.06 were established, respectively. The results indicated the up-regulation of *TGM2* and *HMGA2* in HCT116/OX-R10 cells with high OX-RI and down-regulation of *FXYD3*, *LGALS4*, and *ECI2* in both cell types. Based on the results, *TGM2*, *HMGA2*, *FXYD3*, and *LGALS4* genes are related to oxaliplatin-resistant CRC and may serve as novel therapeutic targets.

## Introduction

Colorectal cancer (CRC) is regarded as the third most commonly diagnosed cancer and the second leading reason for cancer-related mortality worldwide with 1.9 million new cases and 0.9 million deaths during 2020 [1,2]. The CRC treatment strategies include surgery, radiation therapy, chemotherapy, targeted therapy, immunotherapy, and their combination depending on the stage of cancer [3]. Today, cytotoxic chemotherapy is considered as the backbone of CRC treatment [4]. The conventional chemotherapy of CRC contains 5-fluorouracil (5FU) alone or in combination with leucovorin (LV), irinotecan (FOLFIRI), or oxaliplatin (FOLFOX) which are accompanied by the epidermal growth factor receptor (EGFR)- or vascular endothelial growth factor (VEGF)-targeted monoclonal antibodies [5,6]. Chemotherapy, radiotherapy, and targeted therapies only provide a limited overall survival increase, despite advanced screening and treatment in CRC. Resistance to therapy such as chemotherapy and radiotherapy is regarded as the major reason for cancer recurrence and low survival rate. Thus, evaluating the expression levels of critical genes and detecting molecular pathways play a critical role in overcoming the chemoresistance and radioresistance of CRC [7,8].

Oxaliplatin which is considered as an alkylating agent is given for patients with CRC both as first-line chemotherapy and as second-line treatment of 5FU-refractory tumors. The above-mentioned agent can covalently bind to DNA to form intra- and inter-strand platinum-DNA adducts, leading to impaired replication and transcription, as well as cell death [9]. Oxaliplatin-induced DNA damage leads to a transient S-phase and a durable G2/M phase arrest by activating cell cycle checkpoints [10]. Extensive DNA damage and impairment of nucleotide excision repair (NER) increase oxaliplatin-induced apoptosis. The mitogen-activated protein kinase (MAPK) signaling pathway is activated by oxaliplatin, resulting in modulating cell proliferation and survival, as well as apoptosis [11]. Resistance to chemotherapeutic drugs which can be intrinsic or acquired is among the major challenges in CRC therapy. Intrinsic resistance, which is known as primary drug resistance, exists before treatment and patients initially do not respond to therapy. Such resistance can be triggered by the presence of inherent genetic mutations, pre-existing resistant cancer stem cells, and activation of intrinsic pathways involved in detoxification [12,13]. Acquired chemoresistance is developed after therapy and regarded as a progressive and multifactorial phenomenon stemming from complex interactions between intracellular changes and tumor microenvironment such as genetic or epigenetic alterations, increased drug efflux, decreased drug uptake, and mutation or alternation of drug targets [13]. Based on the studies, oxaliplatin resistance may occur through different mechanisms such as drug efflux and detoxification, alterations in oxaliplatin-DNA adducts repair mechanisms, epigenetic modifications such as SRBC epigenetic inactivation, and deregulation of NF-kB signaling pathway. The reasons and potential molecular mechanisms of oxaliplatin resistance remains challenging although some genes such as *OCT2* [14], *ERCC1* [15,16], *MMP7* [17], *WBSCR22* [18] *MUC5AC* [19], HIF-1α [20], and HMGA2 [21] are related to oxaliplatin resistance [17].

Increasing evidence proposed the significance of epithelial-to-mesenchymal transition (EMT) in the tumor progression, metastasis, and drug resistance in the vast majority of cancers during the past decade [22–24]. EMT triggers several alterations in cellular physiology and morphology such as loss of cell polarity, cell–cell adhesion in epithelial cells, gain of motility and migratory properties, as well as degrading and reorganizing the extracellular matrix (ECM) in mesenchymal cells, leading to enhanced cancer invasion [22]. Cells undergoing EMT exhibit enhanced anti-apoptotic factors and drug efflux, as well as decreased stem cells proliferation which confer resistance to anticancer drugs [25,26]. Therefore, targeting genes involved in EMT represent promising strategies to overcome drug resistance.

Using high-throughput “Omics” technologies has led to progress in biomarker discovery, drug development, and target therapy [27]. A number of bioinformatics-based approaches have been developed during the past decade to identify the potential gene biomarkers and metabolic pathways related to cancer from high throughput gene expression data such as microarray and next-generation RNA sequencing (RNA-seq). Microarray data, along with bioinformatics analysis can provide valuable information on complex biological data. Network-based approaches, especially the protein-protein interaction (PPI) network are considered as high-throughput methods for elucidating crucial biological processes and molecular mechanisms in cancer [28,29].

This study seeks to identify and validate the potential genes and pathways related to oxaliplatin resistance in CRC since alterations in genes and mechanisms involved in oxaliplatin resistance are still regarded as unknown. To this aim, differentially expressed genes (DEGs) between the sensitive- and oxaliplatin-resistant CRC cells related to the two identified data series were obtained. Then, the transcriptomics data were analyzed by two approaches because the aforementioned data series differed in terms of oxaliplatin resistance index (OX-RI). In the next step, hub genes were identified through PPI networks. Finally, the mRNA expression level of novel hub genes participating in the regulation of EMT and apoptosis processes, as well as the reported oxaliplatin-resistance genes in the literature were assessed in the parental and established oxaliplatin-resistant HCT116 cells with different OX-RI levels utilizing qRT-PCR.

## Materials and methods

### Bibliographic search and data source

A primary study was conducted applying the GEO database (https://www.ncbi.nlm.nih.gov/geo/) [30] up to April 2023 using the search term [("Colorectal Neoplasms") and ("resistance" or "resistant") and "sensitivity"], which was limited to Homo sapiens in order to achieve microarray expression data of chemoresistant CRC cells. The gene expression datasets were selected utilizing acquired oxaliplatin resistance in the same CRC cell lines, mRNA expression profile, and datasets with biological replicates.

### Detection and extraction of DEGs

Agilent microarray files acquired from the GEO database were evaluated applying GEO2R (https://www.ncbi.nlm.nih.gov/geo/geo2r/) [31] to determine DEGs between the resistant and sensitive groups. Up- and down-regulated genes were extracted by |Log2 fold change (FC)| ≥ 1.5 [32] with a P-value < 0.05 as the cutoff criteria using volcano plot filtering (OriginPro, 2019; OriginLab Corporation, Northampton, MA, USA) (version: b9.6.5.169) [33]. In addition, Venn diagram analysis was performed utilizing OriginPro 2019 to find common significant DEGs between datasets. Further, hierarchical clustering for gene expression data analysis of common genes was conducted applying the pheatmap package (version: 1.0.12) in R (version: 4.3.0) [34].

### PPI network analysis and hub genes screening

The transcriptomics data were examined by two approaches since the above-mentioned data series differed in terms of OX-RI defined as the ratio between the IC_50_ value of resistant cells and that of sensitive cells. Common DEGs in the two datasets were extracted and subset analysis was performed in the first method similar to most published works based on microarray analysis. The DEGs related to oxaliplatin-resistant cells extracted from each GSE were separately investigated for the first time in the second method in order to study the influence of oxaliplatin and OX-RI on mRNA expression of oxaliplatin resistance-related genes. The PPI network of the common DEGs (both up- and down-regulated genes) in the obtained datasets was constructed based on *IMEx* (International Molecular Exchange Consortium; https://www.imexconsortium.org/) and visualized using Cytoscape (https://cytoscape.org/; version: 3.9.1) [35] in order to identify the key genes involved in oxaliplatin resistance-related DEGs independent of RI. IMEx consortium is regarded as a collaboration between major public interaction databases containing IntAct, DIP, HPIDB, MINT, MatrixDB, I2D, InnateDB, UniProtKB/Swiss-Prot, UCL-BHF, and Molecular Connections, which provides a non-redundant set of molecular interactions [36]. Separate PPI networks for each dataset of the achieved up- and down-regulated genes were constructed similarly because the acquired resistance indices of oxaliplatin differed in the selected datasets. The hub genes with high connective interactions (degree ≥ 10) were detected utilizing the CytoHubba plug-in (version: 0.1) [37] in Cytoscape for each network. The hub genes in each dataset were compared with each other. Then, the common hub genes were extracted by Venn diagram analysis. Furthermore, the molecular complex detection (MCODE) plug-in (https://baderlab.org/Software/MCODE/; version: 2.0.2) in Cytoscape was applied in order to identify modules in the whole network with default threshold (degree cutoff: 2; node score cutoff: 0.2; k-core: 2; max. depth: 100; node counts ≥ 4) [38].

### Enrichment analyses for DEGs, hub genes, and modules

Gene ontology (GO) enrichment analysis of the DEGs was implemented by the database for annotation, visualization, and integrated discovery (DAVID) (https://david.abcc.ncifcrf.gov/; version 2021) which is considered as functional annotation database to analyze the DEGs at functional level [39]. Kyoto encyclopedia of genes and genomes (KEGG) (https://www.genome.jp/kegg/; Release 105, 2023) enrichment analysis was applied to obtain an insight into the most enriched pathways of the DEGs [40]. GO terms and KEGG pathways with an EASE score < 0.05 were regarded to be significantly enriched. The functional and pathway enrichment analyses of genes in each module were analyzed by DAVID.

### Cell culture and establishment of oxaliplatin-resistant HCT116 cells

The human CRC HCT116 cell line was purchased from the Research Institute of Biotechnology (Mashhad, Iran) and cultured in Dulbecco’s Modified Eagle Medium (DMEM) high glucose (Capricorn, Germany) with 10% fetal bovine serum (FBS) (Gibco Life Technologies, USA) and 1% penicillin/streptomycin (Biosera, France) at 37°C in 5% CO_2_. Oxaliplatin resistant (HCT116/OX-R) cells were generated by exposing the parental HCT116 cells to the increasing concentrations (0.05–10 μM) of oxaliplatin (Alvoxal, NanoAlvand, Iran) during a 10-month period. The cells were maintained at each drug concentration for 14 days and the medium was refreshed every two days [41,42]. Resistant cells maintained in 4.3 and 10 μM oxaliplatin were named as HCT116/OX-R4.3 and HCT116/OX-R10 cells, respectively.

### Cytotoxicity assay

The in vitro cytotoxicity of oxaliplatin against the parental HCT116 and HCT116/OX-R cells was reviewed using MTT (Sigma, Schnelldorf, Germany) colorimetric assay. In short, the 24 h-cultured cells were treated with increasing concentrations of the drug (0–60 μM) for 48 h. Then, MTT (5 mg mL^-1^) reagent was added into each well. MTT-formazan crystals were dissolved in dimethyl sulfoxide (DMSO) after 4 h incubation and the absorbance was measured at 570 nm utilizing an ELISA reader (BioTek, ELX800, USA). The percentage of cell viability was calculated relative to the untreated control cells. The values of IC_50_ were achieved applying the GraphPad prism software (version 9.5.1). OX-RI was calculated by the following equation.


$$Oxaliplatin-resistance\;index\;(OX-RI)=\frac{IC50\;value\;of\;oxaliplatin-resistant\;cells\;}{IC50\;value\;of\;the\;parental\;cells\;}$$ (1)

### Clonogenic survival assay

Colony formation assay was performed to determine the survival fraction of the parental HCT116 and HCT116/OX-R cells at different concentrations of oxaliplatin. The cells at a density of 100 cells per well were cultured in 24-well plates and treated with increasing concentrations of oxaliplatin (0–60 μM). The culture medium was altered every three days. The cells were fixed with 100% methanol for 5 min after eight days of incubation at 37°C and stained with 0.1% crystal violet for 15 min. The stained colonies were counted under a stereomicroscope, and plating efficiency (P.E.) and survival fraction (S.F.) were calculated as follows.


$$Plating\;Efficiency\;(P.E.)=\frac{Number\;of\;Coloneis}{Number\;of\;cells}$$ (2)

$$Survival\;Fraction\;(S.F.)=\frac{Number\;of\;Colonies\;}{Number\;of\;cells\times P.E.\;}$$ (3)

### Cell cycle analysis

Cell cycle was analyzed by propidium iodide (PI, Sigma, Germany) staining and flow cytometry. The 48-h cultured cells (2 × 10^5^ cells in 6-well plates) were harvested and washed twice with cold phosphate-buffered saline (PBS). Then, single cells were fixed with cold 70% ethanol at 4°C for 24 h, followed by staining with PI solution (10 μg mL^-1^ sodium citrate, 1000 μg mL^-1^ PI, 10 μg mL^-1^ Triton X-100, and 3.3 μg mL^-1^ RNase in PBS). The cell cycle distribution was assessed by flow cytometer (BD Bioscience, Accuri, USA) after one hour incubation in the dark and data were analyzed using FlowJo software (version 7.6.1).

#### Senescence-associated β galactosidase (SA-β-Gal) assay

Senescence-associated β-galactosidase (SA-β-gal) is utilized to detect cellular senescence based on increased levels of beta-galactosidase activity. The cells at a density of 3.2×10^3^ cells per well were grown in 96-well plates until approximately 70% confluent. Then, the cells were fixed with 3.7% formaldehyde for 5 min at room temperature, followed by three times washing with PBS. In the next step, the cells were incubated in staining solution containing 5 mM potassium ferrocyanide, 5 mM potassium ferricyanide, 150 mM NaCl, 20 mM MgCl_2_, 1 mg mL^-1^ X-gal, and 40 mM citric acid/sodium phosphate buffer (pH 6.0) in the dark for 12 h at 37°C. The cells were viewed under an inverted microscope (LABOMED iVu3000-TCM400, Germany) at 200× magnification after washing with PBS.

### Quantitative real-time PCR (qRT-PCR)

Total RNA from sensitive and resistant HCT116 cells was extracted applying a total RNA extraction kit (Roche Applied Sciences, Indianapolis, USA) based on the manufacturer instructions. The quality and quantity of extracted RNA were verified using 1% agarose gel electrophoresis and spectrophotometric analysis at 230, 260, and 280 nm (Thermo Fisher Scientific NanoDrop 2000c, Germany). Then, 1 μg RNA was transcribed into cDNA with random hexamers, oligo dT primers, and H-minus MMLV reverse transcriptase utilizing Reverse Transcription kit (Norgen Biotek Corp, Canada) and the genes of interest were quantified through qRT-PCR in a final volume of 10 μL containing 5 μL of SYBR Green PCR master mix (2x) (Ampliqon, Denmark), 2 μL of primers (10 pmol), 1 μL of cDNA, and 2 μL of nuclease-free water. *Glyceraldehyde-3 phosphate dehydrogenase* (*GAPDH*) and β-actin (*ACTB*) were applied as reference genes. PCR was performed on a Rotor-Gene 6000 thermal Cycler (Corbett Life Science, Germany). Table 1 indicates primer sequences and annealing temperatures. All of the experiments were performed in technical and biological triplicate and gene expression data were normalized to *ACTB* and *GAPDH* by geometrical average. The relative gene expression was calculated using the 2^−ΔΔCT^ method.

**Table 1:** Gene names, accession numbers, primer sequences, and annealing temperatures used in qRT-PCR.

Gene names	Accession number	Primer sequence (5^ʹ^ → 3^ʹ^)	Amplicon size	Annealing temperature (°C)
***GAPDH***	NM_002046	F: GCAGGGGGGAGCCAAAAGGGT R: TGGGTGGCAGTGATGGCATGG	219	58
***BACT***	NM_001101	F: GAGACCTTCAACACCCCAGCC R: AGACGCAGGATGGCATGGG	161	60
***FXYD3***	NM_005971	F: CTCTCATCACCCCAGGCTCAG R: CAAAGGAGTCCCAGCAGAGGA	148	64
***LGALS4***	NM_006149	F: CCACCTACAACCCGACGCT R: AGAACCGCTTCATGTGCTCGCT	109	62
***USH1C***	NM_005709	F: TGTCCATCAAAGTGAGACACATC R: CCCAGATTCCGACACAAAC	101	65
***ECI2***	NM_006117	F: GAAGCTAAAACTCTACGCGC R: AAGGACTCAAACTGGACACC	179	58
***TGM2***	NM_004613	F: GGTGACAAGAGCGAGATGATC R: CACAGCAGTACGTCCCTTCG	142	58
***HMGA2***	NM_003483	F: CACTTCAGCCCAGGGACAAC R: TCTTTTGAGCTGCTTTAGAGGGAC	165	61

GAPDH: Glyceraldehyde-3-phosphate dehydrogenase, BACT: Beta-actin, FXYD3: FXYD domain containing ion transport regulator 3, LGALS4: Lectin galactoside-binding soluble 4, USH1C: Usher syndrome 1C, ECI2: Enoyl-coA delta isomerase 2, TGM2: Transglutaminase 2, HMGA2: High mobility group AT-hook 2, F: Forward, R: Reverse.

### Statistical analysis

All of the data were expressed as the mean ± standard deviation (SD). The statistical comparisons were performed utilizing Student’s t-test and one-way ANOVA were applied for comparing two and three or more groups using GraphPad Prism 9.5.0 1 (GraphPad Software, LLC). The p-values less than 0.05 were considered as statistically significant.

## Results

### Selecting datasets and identifying DEGs

**Fig 1:**
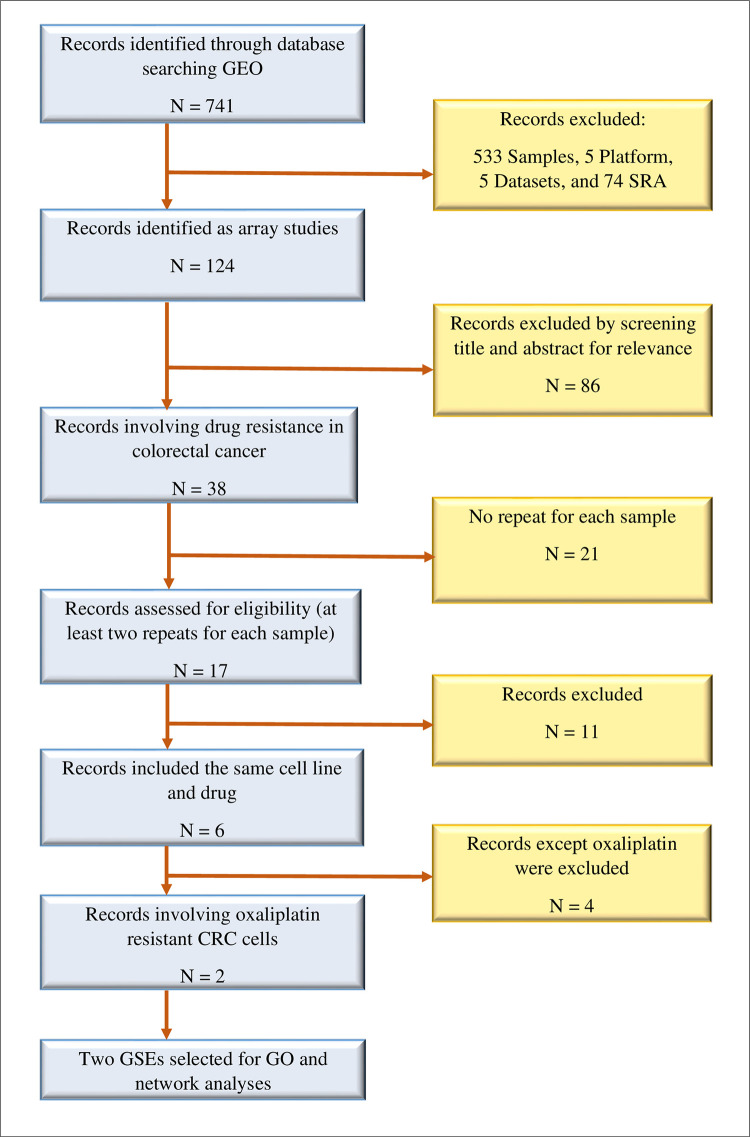
Datasets selection procedure for bioinformatic analysis. Totally, two datasets out of the 741 records identified from the gene expression omnibus (GEO) database meet the selection criteria.

Fig 1 shows the detailed process of literature retrieval. Totally, 741 records were identified through GEO database screening, and six microarray datasets were recognized based on the criteria indicated in the materials and methods section. There are two datasets which compared three replicates of gene expression profiles of oxaliplatin-resistant and susceptible CRC cells, and their GEO accession numbers included GSE42387 and GSE76092.

The microarray data of GSE42387 are based on GPL16297 platform (Agilent-014850 Whole Human Genome Microarray 4x44K G4112F), and the OX-RI value equals 107 [43]. The array data for GSE76092 are based on GPL2125 platform (HumanV2GE 8x60k Microarrays, Agilent design ID: 039494 (original reference: GPL19775 and GPL19773)), and the OX-RI value equals 4.61 [44]. Totally, six samples were obtained for the following analysis, which contained three acquired oxaliplatin-resistant HT-29 cells and three oxaliplatin-sensitive HT-29 samples. The first step focused on identifying the DEGs between the aforementioned data series using GEO2R.

**Fig 2:**
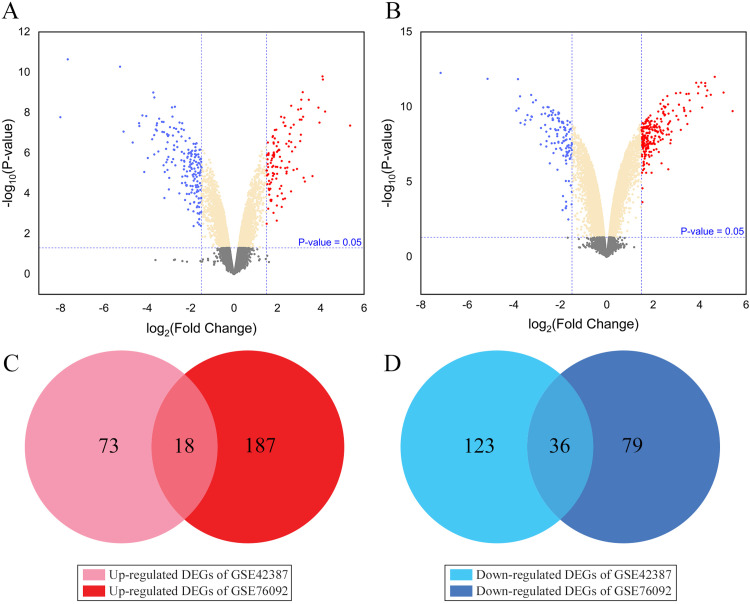
Identifying DEGs between oxaliplatin-resistant and oxaliplatin-sensitive HT-29 cells. Diagrams A and B display the volcano plots related to DEGs for dataset GS4E2387 (A) and GSE76092 (B). The red and blue points represent up- and down-regulated genes, respectively, based on│Log2 FC│≥ 1.5 and adjusted P-value < 0.05. Diagrams C and D demonstrate the Venn diagrams of the 18 up- and 36 down-regulated overlapping DEGs between two data series, respectively.

Volcano plots were utilized for visualizing the DEGs variation between the two GSE datasets (Fig 2A and 2B). There are 250 DEGs (91 up- and 159 down-regulated) in GSE42387 and 320 DEGs (205 up- and 115 down-regulated) in GSE76092 based on described cutoff criteria. Venn diagram analysis displayed 54 common DEGs between two datasets, out of which 18 genes were regarded as up- and 36 ones were considered as down-regulated (Fig 2C and 2D).

**Fig 3:**
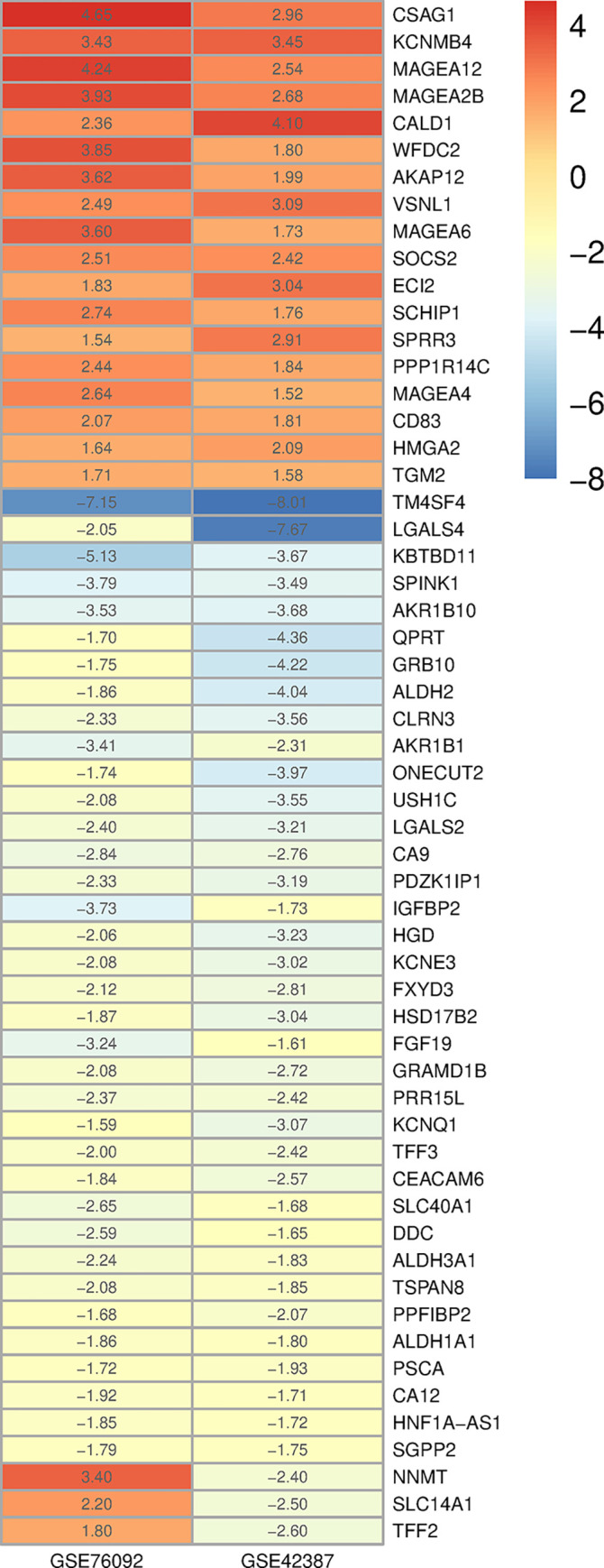
Heatmap related to the log2 FC values of common DEGs between GSE42387 and GSE76092. Each column and row represents a root sample and a gene, respectively. Log2 FC values are shown in each pixel. Red and blue pixels indicate log2 FC ≥ 1.5 and log2 FC ≤ -1.5, respectively. FC: Fold change; DEG: Differentially expressed gene.

**Fig 4:**
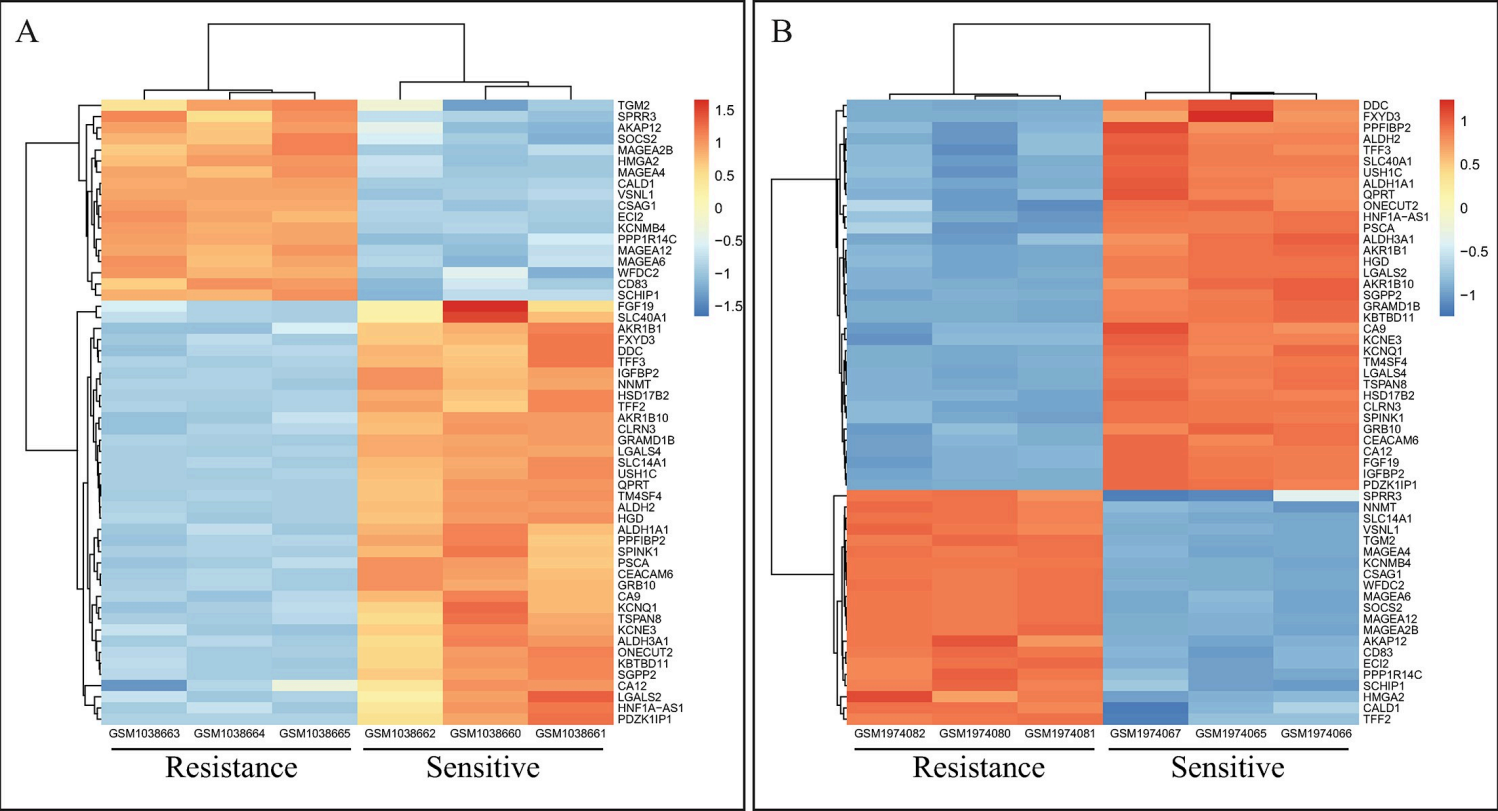
Hierarchical clustering heatmap related to overlapping DEGs. Heatmap related to the expression profile of 57 common DEGs between three resistant and three sensitive HT-29 cells from the microarray (A) GSE42387 and (B) GSE76092 datasets. Red and blue pixels indicate the over- and under-expression of NNMT, SLC14A1, and TFF2 genes in oxaliplatin-resistant cells, respectively. The above-mentioned genes exhibit reverse expression pattern in two datasets to down-regulate in GSE42387 and up-regulate in GSE76092. DEG: Differentially expressed gene.

In addition, three common DEGs including *NNMT*, *SLC14A1*, and *TFF2* were down-regulated in GSE42387 and up-regulated in GSE76092 based on log2 FC values (Fig 3). Hierarchical clustering analysis revealed the expression pattern of common DEGs between the sensitive- and resistant-oxaliplatin cells (Fig 4), indicating that the expression level of the above-mentioned genes in GSE42387 differs from those in GSE76092.

### PPI network construction and modules analysis

**Fig 5:**
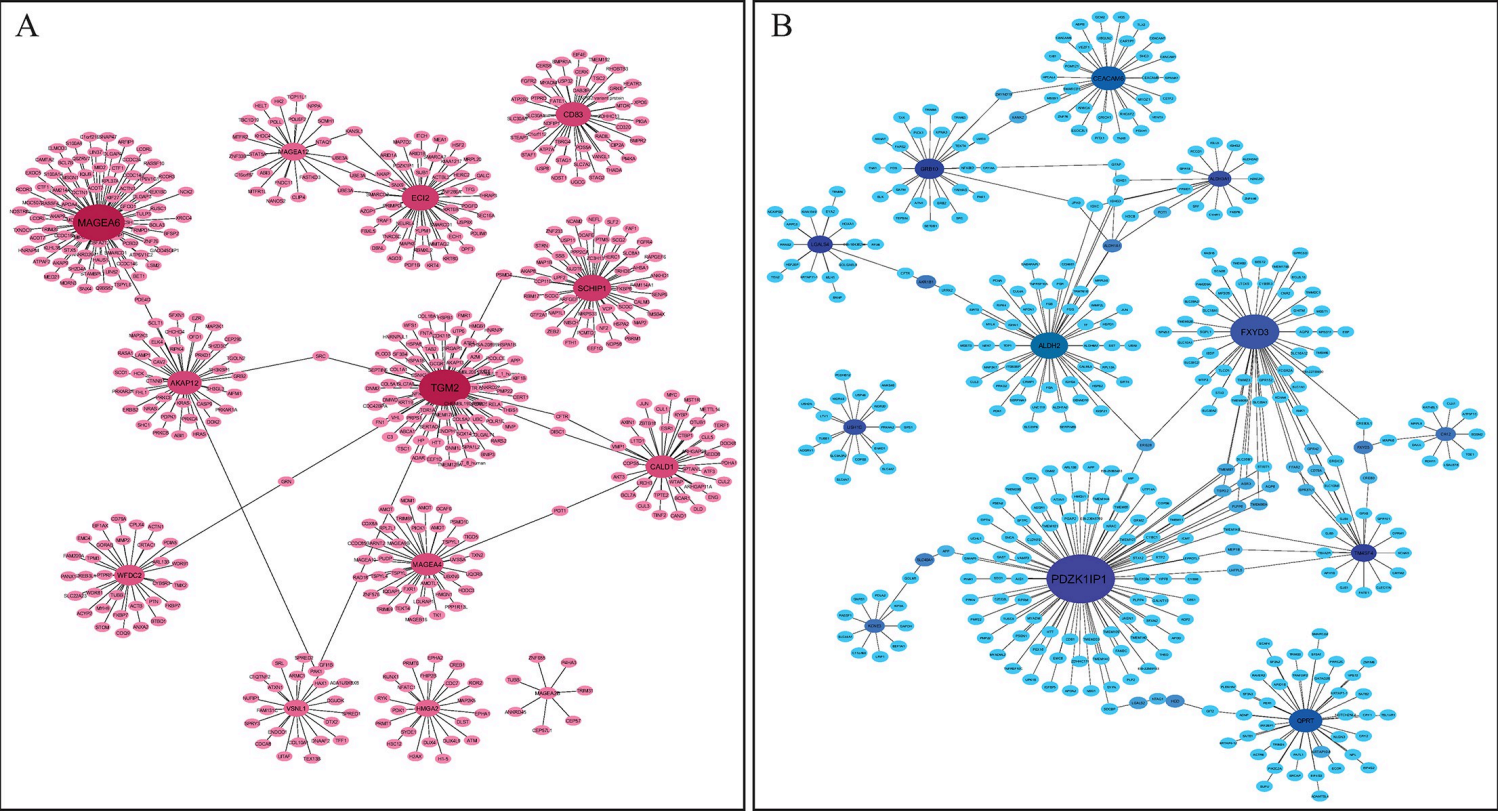
PPI networks of common hub genes in GSE42387 and GSE76092 datasets constructed using the cytoHubba plug-in in Cytoscape based on IMEx database. (A) Up-regulated and (B) down-regulated common DEGs. The nodes and edges represent genes and their interaction, respectively. The red and blue nodes represent the up- and down-regulated DEGs, respectively. The node size and color indicate the degree value. DEG: Differentially expressed gene.

To characterize the expression pattern of genes related to oxaliplatin resistance in CRC cells, the retrieved up- and down-regulated genes of DEGs were separately analyzed to construct the PPI networks utilizing IMEx database for the DEGs of each dataset and the common DEGs between the two datasets. Then, the PPI networks were visualized by the Cytoscape software. PPI network for the common up-regulated DEGs displayed 563 nodes and 655 edges, while 462 nodes and 484 edges were observed in the PPI network related to common down-regulated DEGs (S1 Fig). In the next step, 13 up- and 12 down-regulated hub genes were found in the PPI network related to common DEGs based on degree ≥ 10 (Fig 5). Connectivity degree is calculated based on the number of connections from one gene to the others and the related degree of interaction. *PDZK1IP1* (*PDZK1-interacting protein 1*) which is known as *MAP17* (*membrane-associated protein 17*) and *MAGEA6* exhibit the highest node degree in the down- and up -regulated hub genes, respectively. No module is extracted from the PPI networks related to common DEGs by the Cytoscape MCODE plug-in.

**Fig 6:**
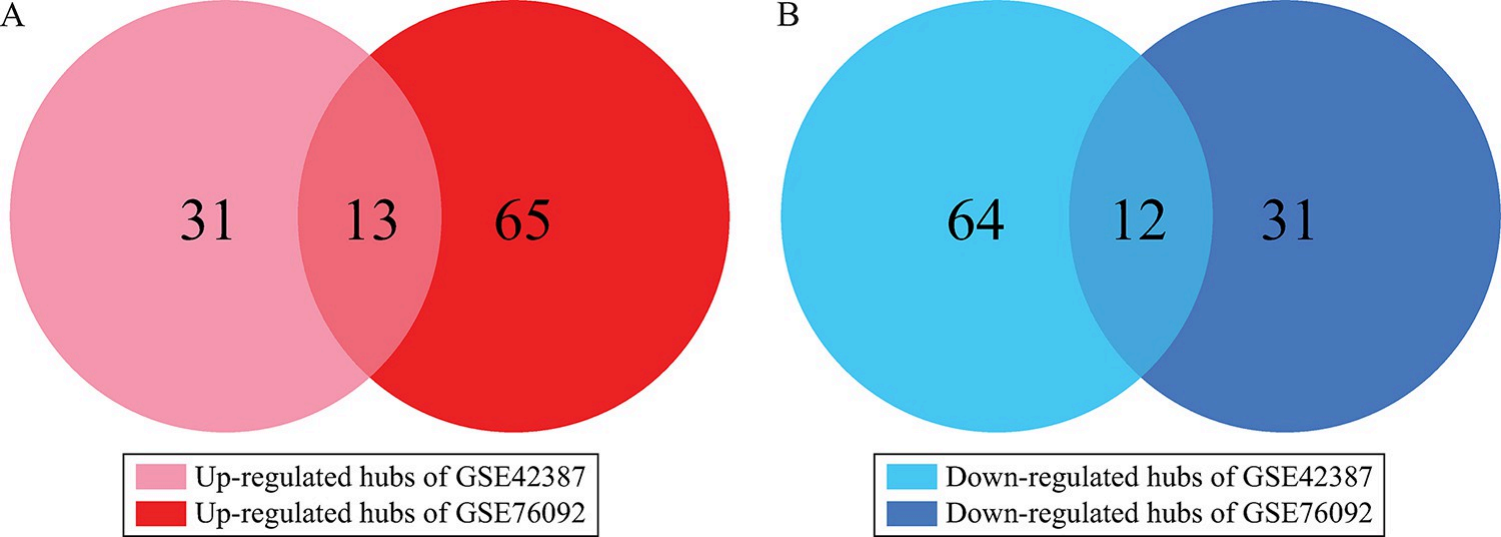
Venn diagrams of overlapping hub genes between GSE42387 and GSE76092. Totally, 25 overlapping hub genes are identified including (A) 12 down-regulated and (B) 13 up-regulated genes. DEG: Differentially expressed gene.

In addition, the PPI networks for the up- and down-regulated DEGs related to each dataset (GSE42387 and GSE76092) were constructed. Totally, 2088 nodes and 2051 edges were involved in the PPI network related to up-regulated genes in GSE42387 and the down-regulated network included 2112 nodes and 2271 edges (S2 Fig). The PPI networks related to up- and down-regulated genes of GSE42387 contained 44 and 76 hub genes, respectively (S1 Table). However, the up- and down-regulated networks of GSE76092 included 3940 nodes and 4346 edges, and 1945 nodes and 2043 edges, respectively (S3 Fig). Totally, 78 up- and 43 down-regulated hub genes were extracted in the PPI networks related to GSE76092 (S1 Table). In addition, 13 up- and 12 down-regulated hub genes were overlapped in the two datasets. The aforementioned hub genes simulated the common hub DEGs in the PPI network (Fig 6, Tables 2 and 3). Other hub genes which were regarded as unique in the above-mentioned datasets were defined as uncommon genes. Tables 4 and 5 show the top 10 up- and 10 down-regulated uncommon hub genes in the two data series.

**Table 2:** The overlapping hub genes in the PPI networks of down-regulated differentially expressed genes (DEGs) from GSE76092 and GSE42387.

Gene symbol	Name Gene	Average Log2 FC	Degree
***PDZK1IP1***	PDZK1 interacting protein 1	-2.8	90
***FXYD3***	FXYD domain containing ion transport regulator 3	-2.5	57
***ALDH2***	Aldehyde dehydrogenase 2 family (mitochondrial)	-3.0	51
***QPRT***	Quinolinate phosphoribosyltransferase	-3.0	39
***CEACAM6***	Carcinoembryonic antigen-related cell adhesion molecule 6	-2.2	31
***GRB10***	Growth factor receptor-bound protein 10	-3.0	26
***TM4SF4***	Transmembrane 4 L six family member 4	-7.6	22
***LGALS4***	Lectin, galactoside-binding soluble 4	-4.9	16
***ALDH3A1***	Aldehyde dehydrogenase 3 family, member A1	-2.0	16
***USH1C***	Usher syndrome 1C	-2.8	16
***KCNE3***	Potassium voltage-gated channel, Isk-related family, member 3	-2.6	10
***CA12***	Carbonic anhydrase XII	-1.8	10

**Table 3:** The overlapping hub genes in the PPI networks of up-regulated differentially expressed genes (DEGs) from GSE76092 and GSE42387.

Gene symbol	Name Gene	Average Log2 FC	Degree
***MAGEA6***	Melanoma antigen family A, 6	2.7	79
***TGM2***	Transglutaminase 2	1.6	78
***SCHIP1***	Schwannomin interacting protein 1	1.9	49
***ECI2***	Enoyl-coa delta isomerase 2	2.4	47
***CD83***	CD83	1.93	43
***AKAP12***	A kinase (PRKA) anchor protein 12	2.7	41
***CALD1***	Caldesmon 1	3.2	38
***MAGEA4***	Melanoma antigen family A, 4	2.1	37
***WFDC2***	WAP four-disulfide core domain 2	2.8	32
***MAGEA12***	Melanoma antigen family A, 12	3.4	24
***VSNL1***	Visinin-like 1	2.8	21
***HMGA2***	High mobility group AT-hook 2	1.9	21
***MAGEA2B***	Melanoma antigen family A, 2B	3.3	10

**Table 4:** The top 10 uncommon hub genes in the PPI networks of up- and down-regulated differentially expressed genes (DEGs) from GSE42387.

Gene symbol	Name Gene	Degree
**Upregulated**		
***KRTAP3-1***	Keratin associated protein 3–1	*88*
***HTR7***	5-hydroxytryptamine receptor 7	*76*
***COL8A1***	Collagen type VIII alpha 1 chain	*73*
***TUBB2B***	Tubulin beta 2B class iib	*62*
***KRT6A***	Keratin 6A	*55*
***KRT6B***	Keratin 6B	*53*
***MID1***	Midline 1	*51*
***OXCT1***	3-oxoacid coa-transferase 1	*48*
***MSN***	Moesin	*41*
***TRIB2***	Tribbles pseudokinase 2	*40*
**Downregulated**		
***CFTR***	CF transmembrane conductance regulator	*212*
***DAPK1***	Death associated protein kinase 1	*169*
***AGR2***	Anterior gradient 2	*102*
***MUC1***	Mucin 1	*96*
***EEF1A2***	Eukaryotic translation elongation factor 1 alpha 2	*87*
***KRT18***	Keratin 18	*87*
***GOPC***	Golgi associated PDZ and coiled-coil motif containing	*84*
***PTK6***	Protein tyrosine kinase 6	*75*
***CASK***	Calcium/calmodulin dependent serine protein kinase	*68*
***ID2***	Inhibitor of DNA binding 2	*65*

**Table 5:** The top 10 uncommon hub genes in the PPI networks of up and down-regulated differentially expressed genes (DEGs) from GSE76092.

Gene symbol	Name Gene	Degree
**Upregulated**		
***MID2***	Midline 2	*259*
***DBN1***	Drebrin 1	*244*
***MKRN3***	Makorin ring finger protein 3	*164*
***PDLIM7***	PDZ and LIM domain 7	*145*
***CAV1***	Caveolin 1	*120*
***ADRB2***	Adrenoceptor beta 2	*99*
***HOMER3***	Homer scaffold protein 3	*88*
***P4HA2***	Prolyl 4-hydroxylase subunit alpha 2	*72*
***KIFAP3***	Kinesin associated protein 3	*69*
***MAGEA1***	MAGE family member A1	*59*
**Downregulated**		
***TRIM54***	Tripartite motif containing 54	*280*
***ANG***	Angiogenin	*120*
***AHNAK***	AHNAK nucleoprotein	*88*
***H1-0***	H1.0 linker histone	*72*
***LGALS9C***	Galectin 9C	*47*
***GJB1***	Gap junction protein beta 1	*45*
***NDRG1***	N-myc downstream regulated 1	*37*
***MVP***	Major vault protein	*37*
***DHRS2***	Dehydrogenase/reductase 2	*35*
***ASNS***	Asparagine synthetase	*33*

Based on the criteria, one functional module from the PPI network related to the down-regulated DEGs of GSE42387 and two ones in the up-regulated DEGs of PPI network related to GSE76092 are identified (Table 6).

**Table 6:** Modules identified from the PPI networks of down- and up-regulated differentially expressed genes (DEGs) from GSE42387 and GSE76092 by MCOD.

	Module	Term
**GSE42387**	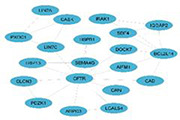 Cluster1	GO: protein localization to basolateral plasma membrane
		GO: maintenance of epithelial cell apical/basal polarity
		GO: Arp2/3 complex-mediated actin nucleation
		GO: neurotransmitter secretion
		GO: plasma membrane
		GO: cell-cell junction
		GO: focal adhesion
		GO: PDZ domain binding
		GO: ATP binding
**GSE76092**	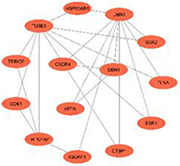 Cluster1	GO: ubiquitin-dependent protein catabolic process
		GO: protein ubiquitination
		GO: negative regulation of apoptotic process
		GO: positive regulation of transcription, DNA-templated
		GO: protein binding
		GO: cadherin binding
		KEGG: hsa05200: Pathways in cancer
		KEGG: hsa05205: Proteoglycans in cancer
		KEGG: hsa05132: Salmonella infection
	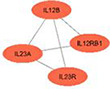 Cluster2	GO: positive regulation of T-helper 17 cell lineage commitment
		GO: positive regulation of memory T cell differentiation
		GO: positive regulation of NIK/NF-kappaB signaling
		GO: positive regulation of inflammatory response
		GO: interleukin-12 receptor binding
		GO: cytokine binding
		KEGG: hsa05321: Inflammatory bowel disease
		KEGG: hsa04630: JAK-STAT signaling pathway
		KEGG: hsa04060: Cytokine-cytokine receptor interaction
		KEGG: hsa05200: Pathways in cancer

The red and blue circles represent up- and down-regulated DEGs, respectively.

### GO and pathway analysis of DEGs

Enrichment analysis was independently performed for the up- and down-regulated common DEGs to find the most essential GO terms, which were affected by oxaliplatin-resistance in CRC cells independent of the value of OX-RI. Detailed GO and pathway analyses of common DEGs are presented in Tables 7–9 and ranked by p-value from low to high (p < 0.05). Based on the GO analysis for the up-regulated genes related to the common DEGs, a significant relationship was observed between the genes in biological process terms and negative regulation of transcription from RNA polymerase II promoter, negative regulation of the apoptotic process, and peptide cross-linking. Histone deacetylase and protein binding in the molecular function were considered as significant (Table 7).

**Table 7:** Functional enrichment analysis of common up-regulated differentially expressed genes (DEGs) in GSE76092 and GSE42387.

Category	Term	Count	P-value	Genes
**GOTERM_BP**	negative regulation of transcription from RNA polymerase II promoter	5	0.00736	*MAGEA12*, *HMGA2*, *MAGEA2B*, *MAGEA6*, *MAGEA4*
**GOTERM_BP**	Negative regulation of apoptotic process	4	0.00831	*SOCS2*, *TGM2*, *HMGA2*, *MAGEA4*
**GOTERM_BP**	peptide cross-linking	2	0.02452	*SPRR3*, *TGM2*
**GOTERM_MF**	histone deacetylase binding	4	0.000162	*MAGEA12*, *MAGEA2B*, *MAGEA6*, *MAGEA4*
**GOTERM_MF**	Protein binding	16	0.0138	*SPRR3*, *MAGEA12*, *CD83*, *SCHIP1*, *ECI2*, *HMGA2*, *MAGEA2B*, *WFDC2*, *SOCS2*, *AKAP12*, *VSNL1*, *CALD1*, *KCNMB4*, *MAGEA6*, *MAGEA4*, *TGM2*

BP: Biological processes; MF: Molecular function.

**Table 8:** Functional enrichment analysis of common down-regulated differentially expressed genes (DEGs) in GSE76092 and GSE42387.

Category	Term	P-value	Genes
**GOTERM_BP**	retinoid metabolic process	0.00127	*AKR1B10*, *ALDH1A1*, *AKR1B1*
**GOTERM_BP**	retinol metabolic process	0.0035	*AKR1B10*, *ALDH1A1*, *AKR1B1*
**GOTERM_BP**	response to insulin	0.006	*KCNQ1*, *IGFBP2*, *GRB10*
**GOTERM_BP**	negative regulation of voltage-gated potassium channel activity	0.0127	*KCNE3*, *KCNQ1*
**GOTERM_BP**	cellular aldehyde metabolic process	0.0143	*ALDH3A1*, *ALDH1A1*
**GOTERM_BP**	doxorubicin metabolic process	0.0159	*AKR1B10*, *AKR1B1*
**GOTERM_BP**	membrane repolarization during action potential	0.0174	*KCNE3*, *KCNQ1*
**GOTERM_BP**	cellular chloride ion homeostasis	0.017	*KCNE3*, *KCNQ1*
**GOTERM_MF**	retinal dehydrogenase activity	7.72E-05	*AKR1B10*, *ALDH1A1*, *AKR1B1*
**GOTERM_MF**	Oxidoreductase activity	4.66E-04	*ALDH1A1*, *HSD17B2*, *AKR1B1*, *ALDH2*, *ALDH3A1*
**GOTERM_MF**	Aldehyde dehydrogenase (NAD) activity	2.87E-04	*ALDH1A1*, *ALDH2*, *ALDH3A1*
**GOTERM_MF**	Benzaldehyde dehydrogenase (NAD^+^) activity	0.003315	*ALDH1A1*, *ALDH3A1*
**GOTERM_MF**	alcohol dehydrogenase (NADP+) activity	0.0050	*ALDH3A1*, *AKR1B10*
**GOTERM_MF**	Galactoside binding	0.006619	*LGALS4*, *LGALS2*
**GOTERM_MF**	alditol:NADP^+^ 1-oxidoreductase activity	0.02	*AKR1B10*, *AKR1B1*
**GOTERM_MF**	Retinal dehydrogenase activity	0.011555	*ALDH1A1*, *AKR1B10*
**GOTERM_MF**	voltage-gated potassium channel activity	0.021	*KCNE3*, *KCNQ1*
**GOTERM_MF**	Carbonate dehydratase activity	0.022981	*CA12*, *CA9*
**GOTERM_CC**	Extracellular exosome	6.8E-05	*PSCA*, *TFF3*, *TSPAN8*, *ALDH1A1*, *FXYD3*, *IGFBP2*, *QPRT*, *DDC*, *HGD*, *AKR1B1*, *CLRN3*, *ALDH2*, *AKR1B10*, *PDZK1IP1*, *SPINK1*
**GOTERM_CC**	basolateral part of cell	0.0031	*CEACAM6*, *TFF3*, *IGFBP2*, *LGALS4*, *AKR1B1*, *ALDH3A1*, *PPFIBP2*, *SPINK1*
**GOTERM_CC**	Basolateral plasma membrane	0.039646	*KCNQ1*, *SLC40A1*, *CA9*
**GOTERM_CC**	extracellular space	0.027	*LGALS4*, *ALDH3A1*, *PPFIBP2*, *CEACAM6*, *FGF19*, *IGFBP2*, *TFF3*, *AKR1B1*
**GOTERM_CC**	plasma membrane	0.049	*CA12*, *GRAMD1B*, *PSCA*, *KCNE3*, *USH1C*, *SLC40A1*, *LGALS4*, *ALDH3A1*, *TSPAN8*, *FXYD3*, *CEACAM6*, *KCNQ1*, *GRB10*, *CA9*

BP: Biological processes; MF: Molecular function; CC: Cellular component.

**Table 9:** Pathway analysis of common down-regulated differentially expressed genes (DEGs) in GSE76092 and GSE42387.

Common down-regulated DEGs				
Category	Term	Count	P-value	Genes
**KEGG_PATHWAY**	Metabolic pathways	12	1.71E-05	*ALDH1A1*, *QPRT*, *DDC*, *HSD17B2*, *AKR1B1*, *HGD*, *ALDH2*, *AKR1B10*, *ALDH3A1*
**KEGG_PATHWAY**	Tyrosine metabolism	3	0.00216	*DDC*, *HGD*,
**KEGG_PATHWAY**	Glycerolipid metabolism	3	0.0063	*AKR1B1*, *ALDH2*, *AKR1B10*
**KEGG_PATHWAY**	Phenylalanine metabolism	2	0.0307	*DDC*, *ALDH3A1*
**KEGG_PATHWAY**	Nitrogen metabolism	2	0.0326	*CA12*, *CA9*
**KEGG_PATHWAY**	Histidine metabolism	2	0.042	*ALDH2*, *ALDH3A1*
**KEGG_PATHWAY**	Folate biosynthesis	2	0.049	*AKR1B10*, *AKR1B1*

The most common down-regulated DEGs are involved in retinoid metabolic process and retinal dehydrogenase activity, respectively. DEGs in the cellular component category are mainly enriched in extracellular exosome (Table 8). KEGG pathway analysis of common up-regulated DEGs displayed no significant pathway, while down-regulated DEGs were significantly enriched in metabolic pathways (Table 9).

**Fig 7:**
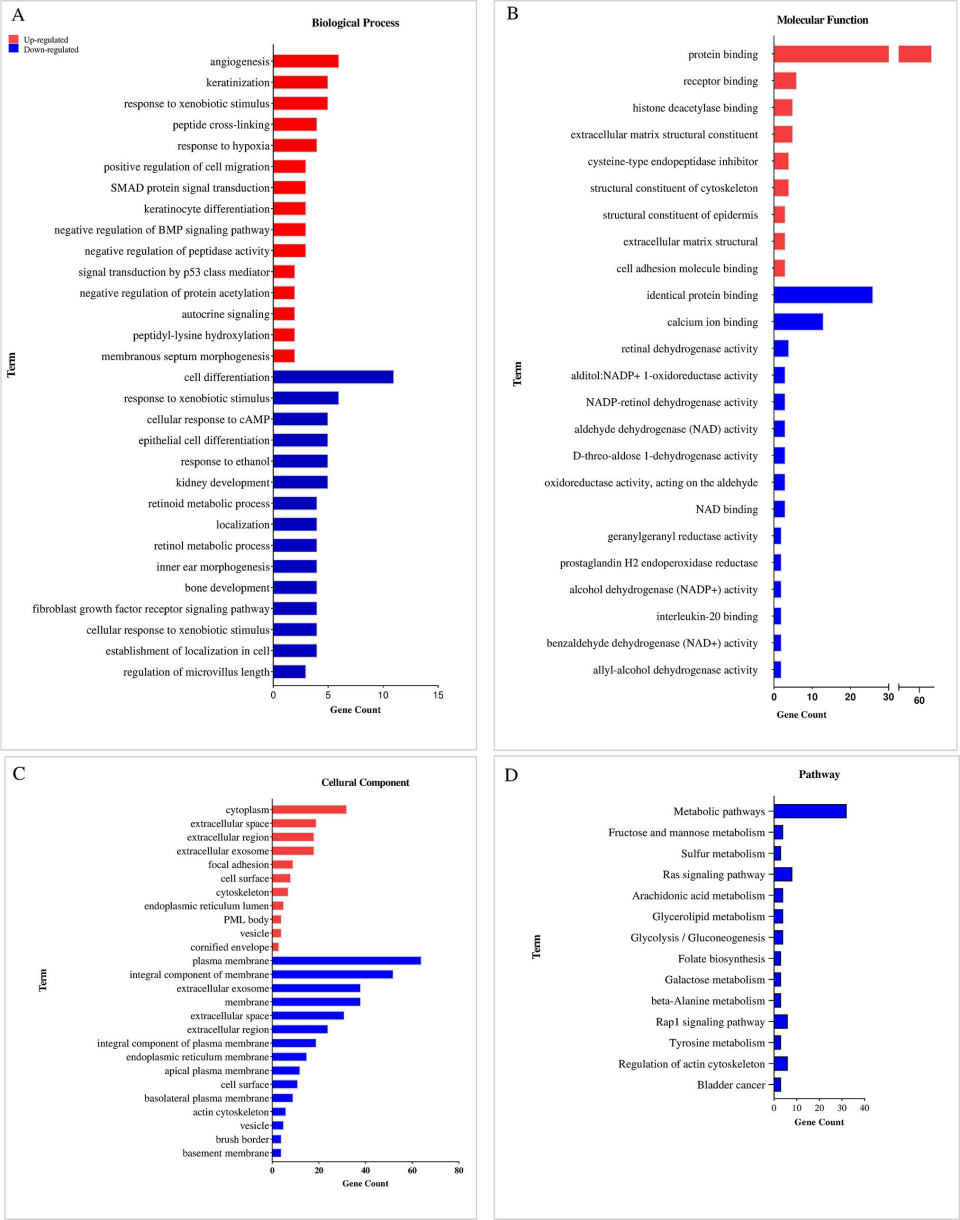
Gene Ontology and pathway analysis of GSE42387 DEGs. The top 15 GO enrichment terms and pathways are shown. (A) Biological process, (B) molecular function, (C) cellular component, and (D) KEGG pathway of up-regulated (red column) and down-regulated (blue column) DEGs. KEGG: Kyoto Encyclopedia of Genes and Genomes; DEGs: Differentially expressed genes. P value is shown in S2 Table.

In addition, GO annotation was performed for the up- and down-regulated DEGs of each dataset based on OX-RI value. The up-regulated DEGs of GSE42387 dataset with an OX-RI value of 107 were involved in the biological process of angiogenesis and in the molecular function of protein binding (Fig 7A and 7B). Cytoplasm is regarded as the most enriched GO term in the cellular component (Fig 7C). Further, cell differentiation, identical protein binding, and plasma membrane are among the most significant GO terms in the biological process, molecular function, and cellular component of the down-regulated DEGs, respectively. Based on the results, no significant pathway was reported for the up-regulated DEGs of GSE42387 and the down-regulated DEGs were enriched in metabolic pathways (Fig 7D).

**Fig 8:**
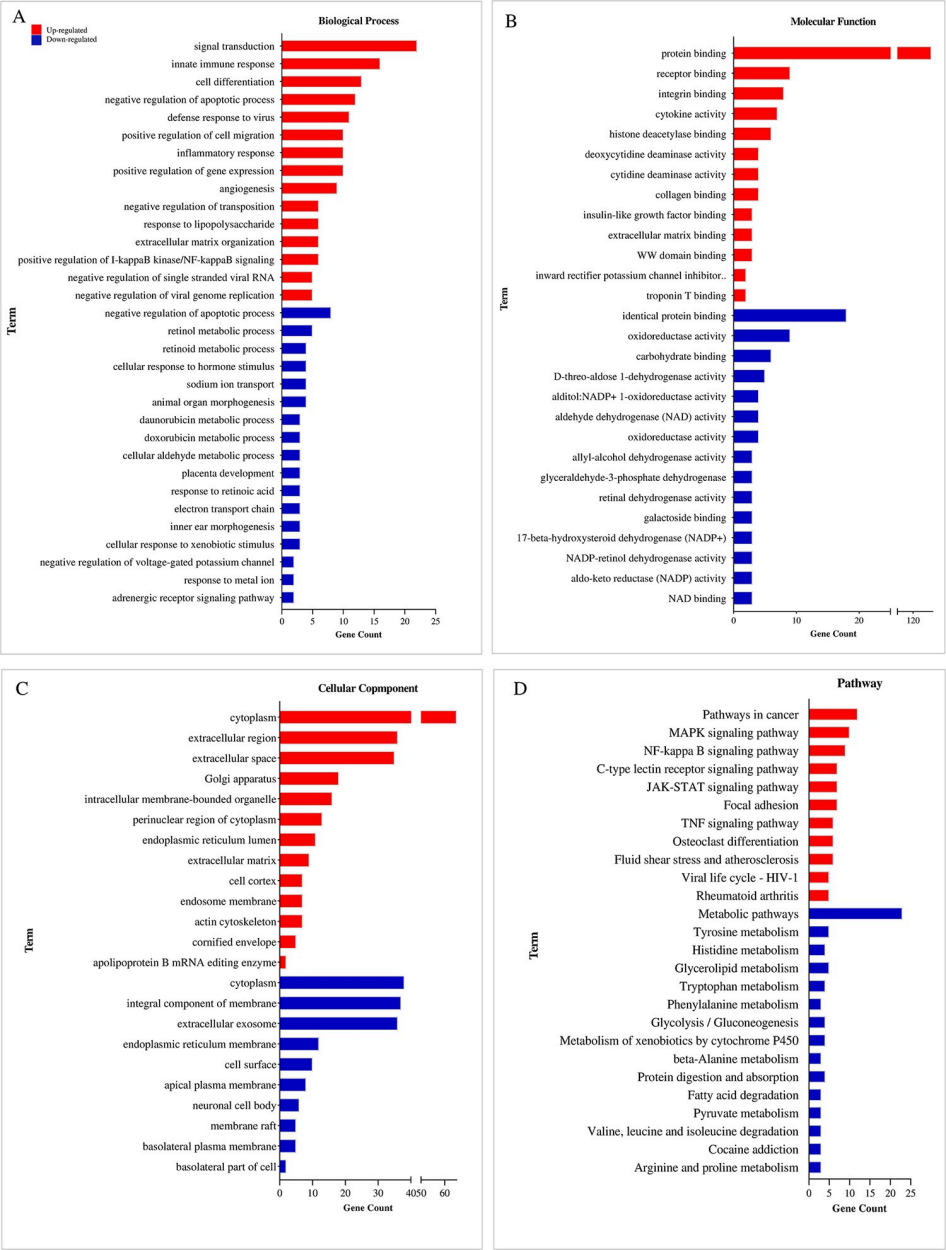
Gene Ontology and pathway analysis of GSE76092 DEGs. The top 15 GO enrichment terms and pathways are shown. (A) Biological process, (B) molecular function, (C) cellular component, and (D) KEGG pathway of up-regulated (red column) and down-regulated (blue column) DEGs. KEGG: Kyoto Encyclopedia of Genes and Genomes; DEGs: Differentially expressed genes. P value is indicated in S2 Table.

The up-regulated DEGs are significantly enriched in signal transduction (biological process), protein binding (molecular function), and cytoplasm (cellular component) in GSE76092 dataset with an OX-RI value of 4.61 (Fig 8A–8C), while biological process, molecular function, and cellular component terms of the down-regulated DEGs are involved in negative regulation of apoptotic process, identical protein binding, and cytoplasm, respectively. As illustrated in Fig 8D, the up-regulated DEGs of GSE76092 are mainly related to pathway in cancer based on KEGG analysis. In addition, metabolic pathways, glycerolipid metabolism, glycolysis/gluconeogenesis, and beta-alanine metabolism are considered as common pathways for the down-regulated DEGs between the two GSE datasets. Table 6 represents GO enrichment analysis of modules in GSE42387 and GSE76092 datasets.

### Establishment and characterization of oxaliplatin-resistant HCT116 cells

**Fig 9:**
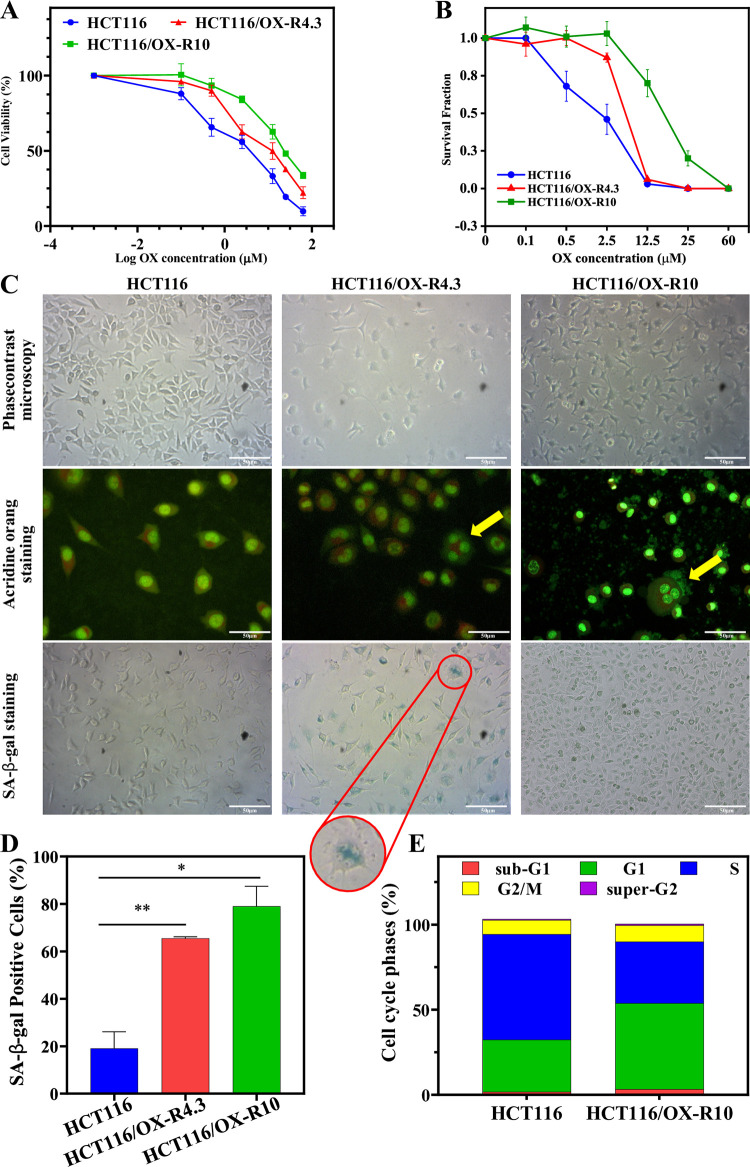
Characterizing oxaliplatin-resistant HCT116 cells. A) Dose-response curves of cell viability in the parental HCT116 cells, HCT116/OX-R4.3, and HCT116/OX-R10 cells treated with various concentrations of oxaliplatin. B) The survival fraction of cells treated with 0.1–60 μM oxaliplatin. C) Representative phase contrast and fluorescence images related to acridine orange- and SA-β-gal—stained parental HCT116, HCT116/OX-R4.3, and HCT116/OX-R10 cells (×20 magnification, Scale bar: 50 μm). Yellow arrows and red circle indicate enlarged multinucleated cells. D) Quantifying the relative percentage of SA-β-gal-positive cells. E) Cell cycle analysis of the parental and resistant HCT116 cells by flow cytometry. Data indicate that the means ± SD of three independent experiment and statistical analysis are performed by one-way ANOVA test. *p < 0.05 and **p < 0.01. HCT116/OX-R: Oxaliplatin-resistant HCT116 cells; SA-β-gal: Senescence-associated beta-galactosidase; OX: Oxaliplatin.

Totally, two oxaliplatin-resistant sub-lines including HCT116/OX-R4.3 and HCT116/OX-R10 cells were derived from HCT116 cells exposed to the increasing concentrations of oxaliplatin until 4.3 and 10 μM, respectively. The IC_50_ value of oxaliplatin for the parental HCT116 cells equals 2.3 ± 0.7 μM, which is significantly lower than for HCT116/OX-R4.3 (9.2 ± 0.4 μM) and HCT116/OX-R10 (23.7 ± 2.4 μM) cells (Fig 9A). The OX-RI value for HCT116/OX-R10 cells is 2.57-fold higher than that of HCT116/OX-R4.3 cells. HCT116/OX-R4.3 and HCT116/OX-R10 cells exhibit significantly higher survival fractions than the parental ones over a range of concentrations 0.1–2.5 μM and 0.1–25 μM of oxaliplatin (Fig 9B) by indicating the less sensitivity of the aforementioned sub-lines to oxaliplatin treatment. As observed in the light microscopic images, morphological alterations in HCT116/OX-R4.3 and HCT116/OX-R10 cells were compared with the parental ones (Fig 9C). Pseudopodia and spindle-like mesenchymal morphology appearing in the resistant cells and their attachment to the surface is regarded as loosen. Based on the acridine orange and SA-β-gal staining, the multinuclear feature and emergence of cellular senescence increase in resistant cells (Fig 9C and 9D). Cell cycle analysis indicated that a greater number of resistant cells are arrested in the G1 phase and fewer cells in the S phase compared to the parental HCT116 cells (Fig 9E). However, no significant alterations were observed in the number of cells in the G2/M phase. The resistant sub-lines exhibited slower growth and more resistance to oxaliplatin.

### Quantitative real-time PCR verification

**Fig 10:**
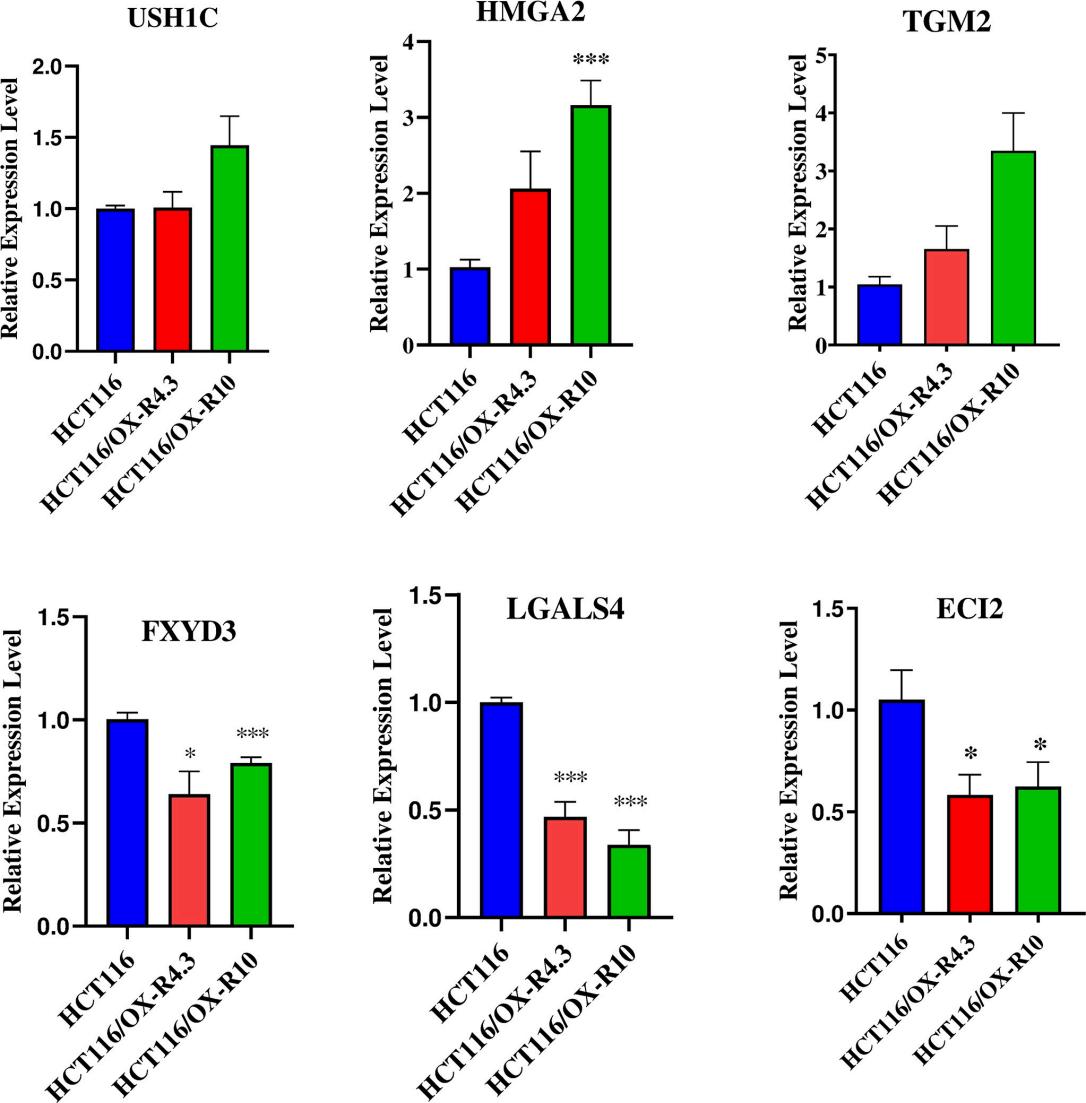
Validating *in silico* analysis by quantitative real-time PCR. Relative mRNA expression levels related to six selected hub genes (*USH1C*, *TGM2*, *HMGA2*, *FXYD3*, *LGALS4*, and *ECI2*) in the parental HCT116 cells and two oxaliplatin-resistant sub-lines, HCT116/OX-R4.3 and HCT116/OX-R10 cells. Data are represented as means ± standard error mean (SEM). Statistical analysis is performed by the student’s t-test compared to the control group; *p < 0.05, and ***p < 0.001. HCT116/OX-R: Oxaliplatin-resistant HCT116 cells; *USH1C*: Usher syndrome 1C; *TGM2*: Transglutaminase 2; *HMGA2*: High mobility group A2 protein; *FXYD3*: FXYD domain-containing ion transport regulator 3; *LGALS4*: Galectin-4; *ECI2*: Enoyl-coA delta isomerase 2.

The qRT-PCR was performed to experimentally validate the expression level of *FXYD3*, *LGALS4*, *USH1C*, *ECI2*, *TGM2*, and *HMGA2* genes in the parental and oxaliplatin-resistant HCT116 cells (Fig 10). Based on the results, FXYD3 and LGALS4 transcripts were down-regulated in HCT116/OX-R4.3 and HCT116/OX-R10 with low and high OX-RI value cells compared to the parental HCT116 cells, while *TGM2* and *HMGA2* were significantly up-regulated only in HCT116/OX-R10 cells. The expression level of *ECI2* decreased in both resistant cells, unlike the microarray data, indicating that such gene is up-regulated (Table 3). No significant difference was reported in the *USH1C* mRNA level between the parental and oxaliplatin-resistant HCT116 cells.

## Discussion

Acquired resistance to chemotherapeutic drugs decreased effectiveness and overall survival, and is considered as the leading reason for death, despite advances in the medical treatment of CRC [45]. Identifying novel hub genes involved in CRC cells resistant to platinum-based agents improves survival rates and avoids cancer recurrence [46]. Thus, the extracted DEGs of oxaliplatin-resistant HT-29 cells from the two datasets of GSE42387 and GSE76092 were analyzed in two different methods to identify the key genes involved in the molecular mechanisms of oxaliplatin-resistance, as well as those related to the RI of CRC cells to oxaliplatin. In addition, two sub-lines resistant to oxaliplatin including HCT116/OX-R4.3 and HCT116/OX-R10 cells were established. The mRNA expression level of *FXYD3*, *LGALS4*, *USH1C*, *ECI2*, *TGM2*, and *HMGA2* genes which are involved in EMT, drug resistance, and cancer progression were measured in HCT116/OX-R4.3 and HCT116/OX-R10 cells by qRT-PCR.

Further, 13 up- (*MAGEA6*, *TGM2*, *SCHIP1*, *ECI2*, *CD83*, *AKAP12*, *MAGEA4*, *CALD1*, *WFDC2*, *MAGEA12*, *VSNL1*, *HMGA2*, and *MAGEA2B*) and 12 down-regulated (*PDZK1IP1*, *FXYD3*, *ALDH2*, *QPRT*, *CEACAM6*, *GRB10*, *TM4SF4*, *LGALS4*, *ALDH3A1*, *USH1C*, *KCNE3*, and *CA12*) hub genes were overlapped between the PPI networks of GSE42387 and GSE76092 datasets. The above-mentioned hub genes were the same as those obtained from the PPI networks of common DEGs. Uncommon (nonoverlapping) hub genes could stem from differences in OX-RI values and resistance pattern considering that the acquired OX-RI values are not identical for the two studies data series. However, the common hubs obtained from the two data series appear to be independent of OX-RI values. Thus, the aforementioned genes can be regarded as putative causal genes of oxaliplatin-resistance in CRC.

Pathway enrichment analysis indicated that common down-regulated DEGs mainly participated in metabolic pathways and glycolysis/gluconeogenesis, fatty acid, and amino acid metabolism. Sun et al. (2019) reported that metabolic pathways and fatty acid catabolic are considered as crucial processes related to drug resistance in lung cancer cells [47]. Based on the results, cancer cells alter their metabolism in response to widely-used first-line chemotherapy which can be tumor- and drug-specific [48]. The results indicated that the down-regulated DEGs in both datasets are enriched in metabolic pathways, glycolysis/gluconeogenesis, as well as glycerolipid, tyrosine, and beta-alanine metabolism. In addition, down-regulated hub genes including aldehyde dehydrogenase (*ALDH*) family, *QPRT*, *AKR1B10*, *AKR1B1*, *HGD*, and *DCC* were common between the pathways of the two data series. The results indicated the down-regulation of *ALDH1A1*, *ALDH2*, *ALDH3A1*, *and QPRT* in both datasets. High ALDH activity is implicated in tumor progression and drug resistance [49]. However, few studies have been conducted on the relationship between the under-expression of the above-mentioned genes in tumors and lower survival. For example, Chang et al. (2018) argued that the down-regulation of *ALDH2* in tumor is related to cancer progression and may derive from cancer metabolism [50]. Quinolinate phosphoribosyl transferase (QPRT) is regarded as a critical enzyme in the catabolism of quinolinate. QPRT affects malignant phenotype in cancer cells and is proposed as an antitumoral gene in prostate cancer which may prevent the progression of the disease [51,52]. There are some reports correlating the elevated levels of QPRT with resistance due to its anti-apoptotic properties [53,54]. However, the function of QPRT in oxaliplatin resistance CRC is regarded as unknown and has not been considered yet. Therefore, the role and mechanism of QPRT and ALDH family in the resistance of CRC to chemotherapeutic drugs should be discussed in future studies.

The up-regulated pathways could not be compared between the two GSE since no significant pathway was found in the up-regulated DEGs of GSE42387. In addition, Angiogenesis, signal transduction by p53 class mediator, and peptide cross-linking were common between both datasets containing high and low oxaliplatin resistance indices. Further, *TGM2* and *MAGEA2B* were common among the aforementioned biological processes.

*PDZK1IP1* and *FXYD3* exhibited the highest degree in the PPI network among the common down-regulated hubs. PDZK1IP1 or MAP17 which is regarded as a small membrane protein is overexpressed in various carcinoma [55]. The high levels of *PDZK1IP1* predict an appropriate response and survival among patients with cervical carcinoma treated with cisplatin and radiotherapy [56]. Thus, the low level of *PDZK1IP1* is probably related to poor survival and chemotherapy resistance. *FXYD3* which is considered as an ion transport regulator is reported to be up- or down-regulated in different types of cancer [57,58]. Jin et al. (2021) claimed that FXYD3 was significantly down-regulated and proposed as a prognostic factor for recurrence in colon cancer samples [59]. FXYD3 is related to cell adhesion and induces EMT via TGF-beta signaling [60]. The down-regulation of FXYD3 in acquired oxaliplatin-resistant CRC HT-29, HCT116/OX-R4.3, and HCT116/OX-R10 cells with different OX-RI values were observed here. Targeting FXYD3 by miR-143 regulated the expression of FXYD3 negatively and its overexpression increased chemo-sensitivity to oxaliplatin in CRC cells [17,61,62]. In addition, the high expression of FXYD3 induced by 5-fluorouracil and oxaliplatin in a p53-functional cell line (MCF-7) was attributed to the binding of p53 to putative elements in FXYD3 [63]. Therefore, examining the expression of miRNAs targeted to FXYD3 and the function of p53 in resistant CRC cells should be regarded in subsequent studies to achieve a better result and overcome the drug resistance.

Other common down-regulated hub genes with a lower degree in PPI network included galectin-4 (LGALS4) and USH1 protein network component harmonin (USH1C), which participate in EMT. EMT-programmed activation critically contributes to the development of therapeutic resistance in multiple cancer types [25]. *LGALS4* is considered as a tumor suppressor in CRC and mediates cell adhesion [64,65]. The decrease in *LGALS4* expression may activate Wnt/β-catenin signaling pathway in cytoplasm [65], leading to survival of tumor cells [66]. The expression level of *LGALS4* in HCT116/OX-R4.3 and HCT116/OX-R10 cells was lower compared to the parental HCT116 ones, indicating its effective role in oxaliplatin resistance which in consistent with the *in silico* results of this study. The down-regulation of *LGALS4* in oxaliplatin-resistant CRC cells may promote EMT, as well as inducing proliferation and migration of resistant cells. In addition, *USH1C* which is regarded as another down-regulated hub gene is enriched during regulating the microvillus length and participates in EMT through loss of microvilli during EMT [67]. Accordingly, LGALS4 and USH1C appear to be related to EMT development and the consequent oxaliplatin-resistance in CRC cells.

The top two nodes in the PPI network related to common up-regulated hub genes include *melanoma-associated antigen family-A*6 (*MAGE-A*6) and *transglutaminase 2* (*TGM2*). A family of cancer/testis antigens called *MAGE*-A is expressed in tumor cells, despite its silence in normal tissues. Some studies reported that *MAGE*-A is significantly overexpressed in CRC cells [68,69] and related to chemoresistance in other types of cancers although their biological function is mostly unknown [70,71]. *TGM2* induces chemoresistance in cancer cells by modulating extracellular matrix structure, inducing EMT, activating survival pathways, especially Wnt/β-catenin signaling, and inhibiting apoptosis and autophagy [72,73]. In addition, the down-regulation of *TGM2* is proposed to serve as a form of CRC and drug resistance therapy strategy [74,75]. The present GO analysis of common DEGs revealed that *TGM2* and *HMGA2* contribute to negative regulation of the apoptotic process. Overexpression of *HMGA2* which is considered as a non-histone architectural transcription factor can contribute to chemoresistance by inducing EMT, senescence, anti-apoptotic, and epigenetic mechanisms [76,77]. Deng et al. (2021) found that the overexpression of *HMGA2* promotes PI3K/Akt activation in oxaliplatin resistance in CRC [21]. Some recent studies indicate an interaction between *HMGA2* and Wnt/β-catenin signaling pathway in CRC [78,79]. Here, the results indicate the up-regulation of *HMGA2* and *TGM2* in oxaliplatin-resistant HT-29 and HCT116/OX-R10 cells, emphasizing the role of the above-mentioned genes in acquiring resistance to oxaliplatin. As indicated, the down-regulation of *LGALS4* results in promoting EMT, decreasing apoptosis, and activating Wnt/β-catenin signaling pathway. Thus, targeting *HMGA2*, *TGM2*, and *LGALS4* genes may be an effective treatment for oxaliplatin-resistant CRC. *LGALS4* exhibited the highest level of under expression in both established oxaliplatin-resistant sub-lines among the aforementioned candidate biomarkers and displayed high fold change value with p-value equal to 7.4E-07 in the two microarray datasets. Therefore, *LGALS4* can be proposed as the most significant potential biomarker here. Accordingly, other three genes as potential markers related to oxaliplatin-resistant CRC in descending order are ranked as *FXYD3*, *HMGA2*, and *TGM2*.

*ECI2* (*enoyl-CoA delta isomerase 2*) and *Schwannomin-interacting protein 1* (*SCHIP1*) genes are reported here for the first time to be involved in drug resistance among common up-regulated hub genes. No evidence is observed in the literature regarding the involvement of the above-mentioned genes in oxaliplatin resistance. *ECI2* and *SCHIP1* genes may probably be regarded as novel genes in oxaliplatin-resistant CRC cells. SCHIP1 protein has been suggested to be involved in schwannomin activity [80] although its function in cancer is considered as unknown. The exact role of *ECI2* in the process of carcinogenesis is regarded as unknown, as well. A few studies have reported that the up-regulation of *ECI2* which is considered as a downstream target of androgen receptor promotes prostate cancer progression [81] and inhibits cell death response through lipid degradation [82,83]. The high expression of *ECI2* was proposed to be a potential therapeutic target in prostate cancer [84]. The results indicated the down-regulation of *ECI2* in both HCT116/OX-R4.3 and HCT116/OX-R10 cells. The difference between *in silico* and *in vitro* expression pattern of *ECI2* can be ascribed to the different cell type investigated and OX-RI values. Other identified common up-regulated hub genes such as AKAP12 [85,86], WFDC2 [87,88], CALD1 [89,90], and VSNL1 [91,92] are related to drug resistance in various cancers although their involvement in oxaliplatin resistance in CRC was not identified and can be reported as protentional biomarkers which merits further exploration.

The role of uncommon hub genes in the studied datasets was examined with different OX-RI values. *COL8A1* and *KRT6B* which are regarded as up-regulated hub genes in GSE42387 dataset with high interaction degree and log2 FC are involved in angiogenesis and cisplatin-resistance, respectively [93,94]. Zhang et al. (2021) asserted that the inhibition of *CFTR* which is considered as the top down-regulated hub gene in GSE42387 dataset promoted EMT phenotypes and facilitated cell migration, invasion, and metastasis in CRC [95]. In addition, GO and pathway analysis of down-regulated genes in module 1 of GSE42387 dataset proved that the decrease in cell adhesion is regarded as a significant process, which affects the occurrence of EMT and drug resistance [25]. Thus, the EMT process can be proposed as a marker of resistance to oxaliplatin in CRC with high OX-RI value. However, the preeminent processes in clusters 1 and 2 of GSE76092) low OX-RI value(, protein ubiquitination and inflammatory response, promote tumor progression and metastasis through antiapoptotic process and NF-κB signaling pathway [83,96]. It is worth noting that EMT during carcinoma invasion and metastasis is considered as reversible and dynamic, and exhibits epithelial and/or mesenchymal phenotype, which is known as partial EMT [25]. Therefore, EMT process may be activated partially in oxaliplatin-resistant CRC with different resistance indices. Overexpression of *MID2* and *DBN1* which are regarded as the top up-regulated hub genes in GSE76092 dataset is related to chemoresistance in breast cancer and leukemia, respectively [97,98]. *AHNAK* serves as a p53 cofactor among the top down-regulated hub genes [99] and its overexpression decreases chemoresistance in breast and lung cancer [100]. Conflicting results are reported throughout the literature regarding the expression of some of uncommon hub genes such as *OXCT1* [101,102], *AGR2* [103,104], *MUC1* [105,106], *GRP78* [107,108], and *TRIM54* [83] in drug-resistant cancer cells. The aforementioned results confirm the hypothesis that resistance index affects gene expression and common hub genes which were independent of OX-RI values exhibit more reliable results. Evaluating the protein levels of hub genes and finding effective transcription and epigenetic factors, as well as miRNA networks can provide critical information to understand drug resistance better.

Limitations in this study should be considered in interpreting the results. The high-throughput data applied for analysis were regarded as limited and only two datasets in the GEO database benefitted from the inclusion criteria. *In silico* validation was performed on the microarray data of oxaliplatin-resistant HT-29 CRC cell line. Thus, using different CRC cell lines containing various OX-RI values and clinical samples are needed to improve the reliability of data. In addition, high throughput omics data should be integrated and analyzed utilizing machine learning approach and molecular biology experiments to validate the results.

## Conclusion

The present study identified a number of hub genes related to the molecular mechanism of oxaliplatin resistance by comprehensive bioinformatics analysis, which may help determine potential gene therapy targets for oxaliplatin-resistant CRC cells. Based on the results, OX-RI value affects the gene expression pattern significantly. Therefore, different levels of drug resistance should be studied to achieve a reliable result. The present data propose the involvement of different pathways and processes, as well as metabolism alterations to facilitate metastasis and oxaliplatin resistance. The results indicated the down-regulation of *LGALS4*, *FXYD3*, and *ECI2* and the up-regulation of *TGM2* and *HMGA2* in HCT116/OX-R10 cells with high OX-RI value. The above-mentioned genes via EMT induction and apoptosis reduction can resist CRC cells to oxaliplatin. Combined targeting therapy strategies involving multiple genes and pathways may achieve better therapeutic responses. This study focused on bioinformatics and the qRT-PCR results only in two acquired oxaliplatin-resistant HCT116 sub-lines with different OX-RI values. Therefore, the conclusion should be confirmed by further studies involving expression profiling in larger sample sizes and *in vivo* experiments. Employing artificial intelligence-based biology approaches combined with network analysis can identify the main pathways involved in metabolic reprogramming during carcinogenesis and help understand drug resistance and cancer recurrence better.

## Supporting information

S1 Fig PPI networks of common differentially expressed genes in GSE42387 and GSE76092 datasets by IMEx database and visualized in cytoscape. The nodes represent genes and edges represent interaction between genes. The red and blue nodes signify the up-regulated and down-regulated DEGs, respectively. The node size and color indicate the degree value. DEG: Differentially expressed gene; PPI: Protein-protein interaction.
(TIF)

S2 Fig PPI networks of differentially expressed genes in GSE42387 dataset by IMEx database and visualized in cytoscape. The nodes represent genes and edges represent interaction between genes. The red and blue nodes signify the up-regulated and down-regulated DEGs, respectively. The node size and color indicate the degree value. DEG: Differentially expressed gene; PPI: Protein-protein interaction.
(TIF)

S3 Fig PPI networks of differentially expressed genes in GSE76092 dataset by IMEx database and visualized in cytoscape. The nodes represent genes and edges represent interaction between genes. The red and blue nodes signify the up-regulated and down-regulated DEGs, respectively. The node size and color indicate the degree value. DEG: Differentially expressed gene; PPI: Protein-protein interaction.
(TIF)

S1 Table Up-regulated and down-regulated hub genes identified from the PPI network of the two GSE datasets. (XLSX)

S2 Table Detailed information of the GO and pathways analysis of the differentially expressed genes of the two GSE datasets. (XLSX)
