# Correction: *TGM2*, *HMGA2*, *FXYD3*, and *LGALS4* genes as biomarkers in acquired oxaliplatin resistance of human colorectal cancer: A systems biology approach

**DOI:** 10.1371/journal.pone.0322319

**Published:** 2025-04-04

**Authors:** Tayebeh Cheraghi-shavi, Razieh Jalal, Zarrin Minuchehr, Frouzandeh Mahjoubi

Frouzandeh Mahjoubi is not included in the author byline. Frouzandeh Mahjoubi should be listed as the fourth author and is affiliated with Department of Medical Genetics, Institute of Medical Biotechnology, National Institute of Genetic Engineering and Biotechnology, Tehran, Iran. The contributions of this author are as follows: Methodology, Supervision.

The correct citation is: Cheraghi-shavi T, Jalal R, Minuchehr Z, Mahjoubi F (2023) *TGM2*, *HMGA2*, *FXYD3*, and *LGALS4* genes as biomarkers in acquired oxaliplatin resistance of human colorectal cancer: A systems biology approach. PLoS ONE 18(8): e0289535. https://doi.org/10.1371/journal.pone.0289535.
