# Supplementary figures and images for: TGM2, HMGA2, FXYD3, and LGALS4 genes as biomarkers in acquired oxaliplatin resistance of human colorectal cancer: A systems biology approach
Source: PLoS One. 2023 Aug 3;18(8):e0289535. doi: 10.1371/journal.pone.0289535 (PMC10399784; doi:10.1371/journal.pone.0289535)

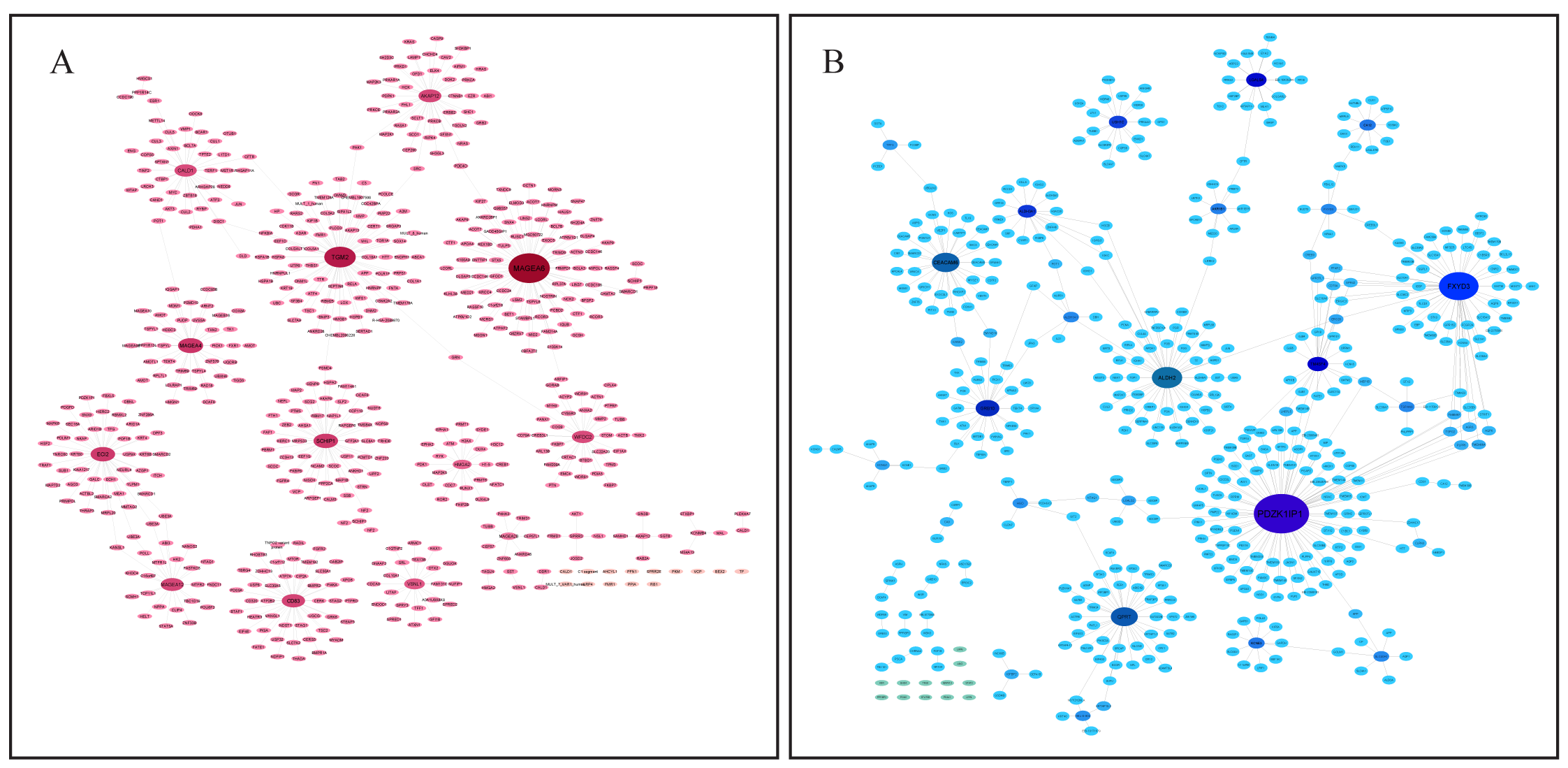

Supplement: S1 Fig — The nodes represent genes and edges represent interaction between genes. The red and blue nodes signify the up-regulated and down-regulated DEGs, respectively. The node size and color indicate the degree value. DEG: Differentially expressed gene; PPI: Protein-protein interaction. (TIF) [file pone.0289535.s001.tif]

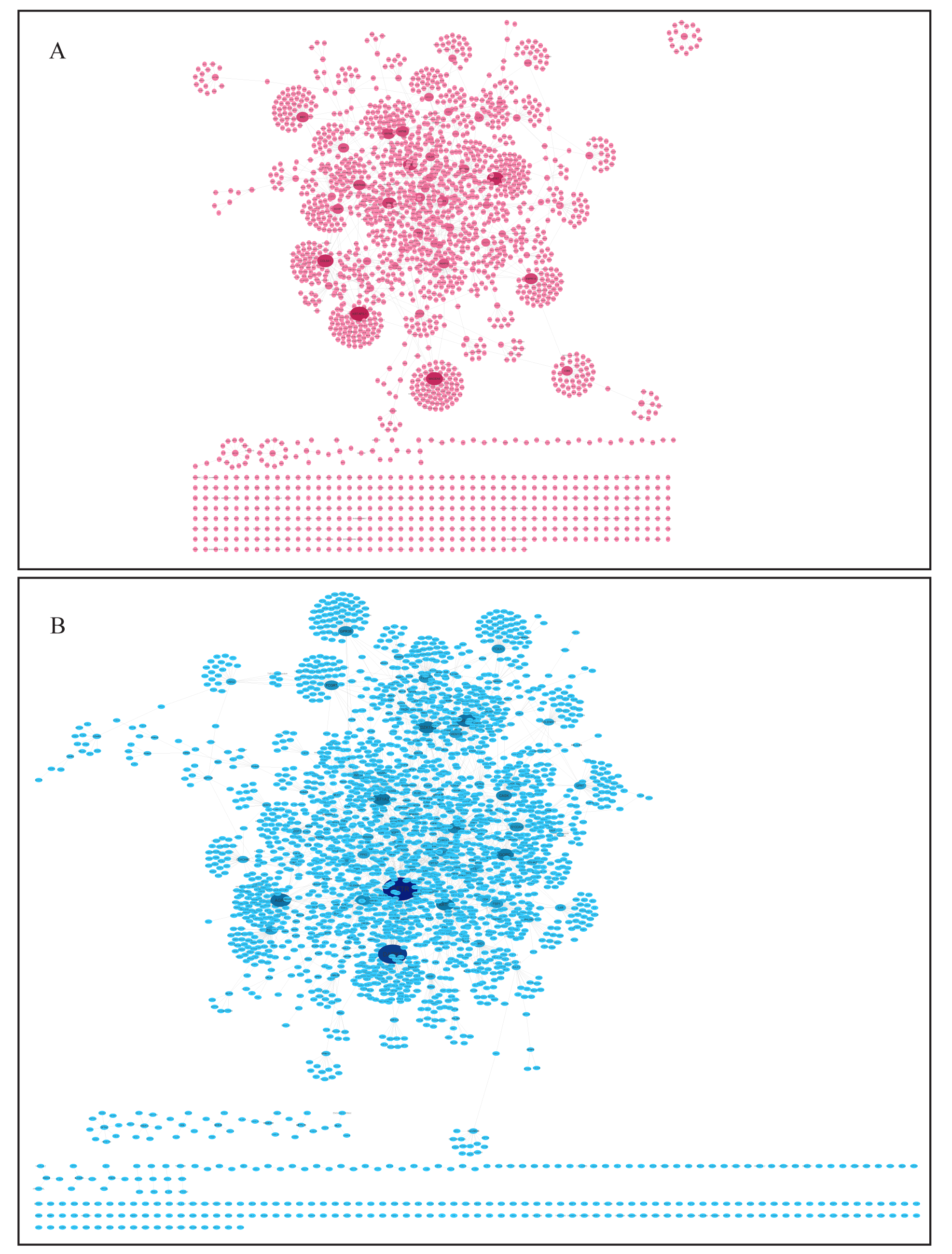

Supplement: S2 Fig — The nodes represent genes and edges represent interaction between genes. The red and blue nodes signify the up-regulated and down-regulated DEGs, respectively. The node size and color indicate the degree value. DEG: Differentially expressed gene; PPI: Protein-protein interaction. (TIF) [file pone.0289535.s002.tif]

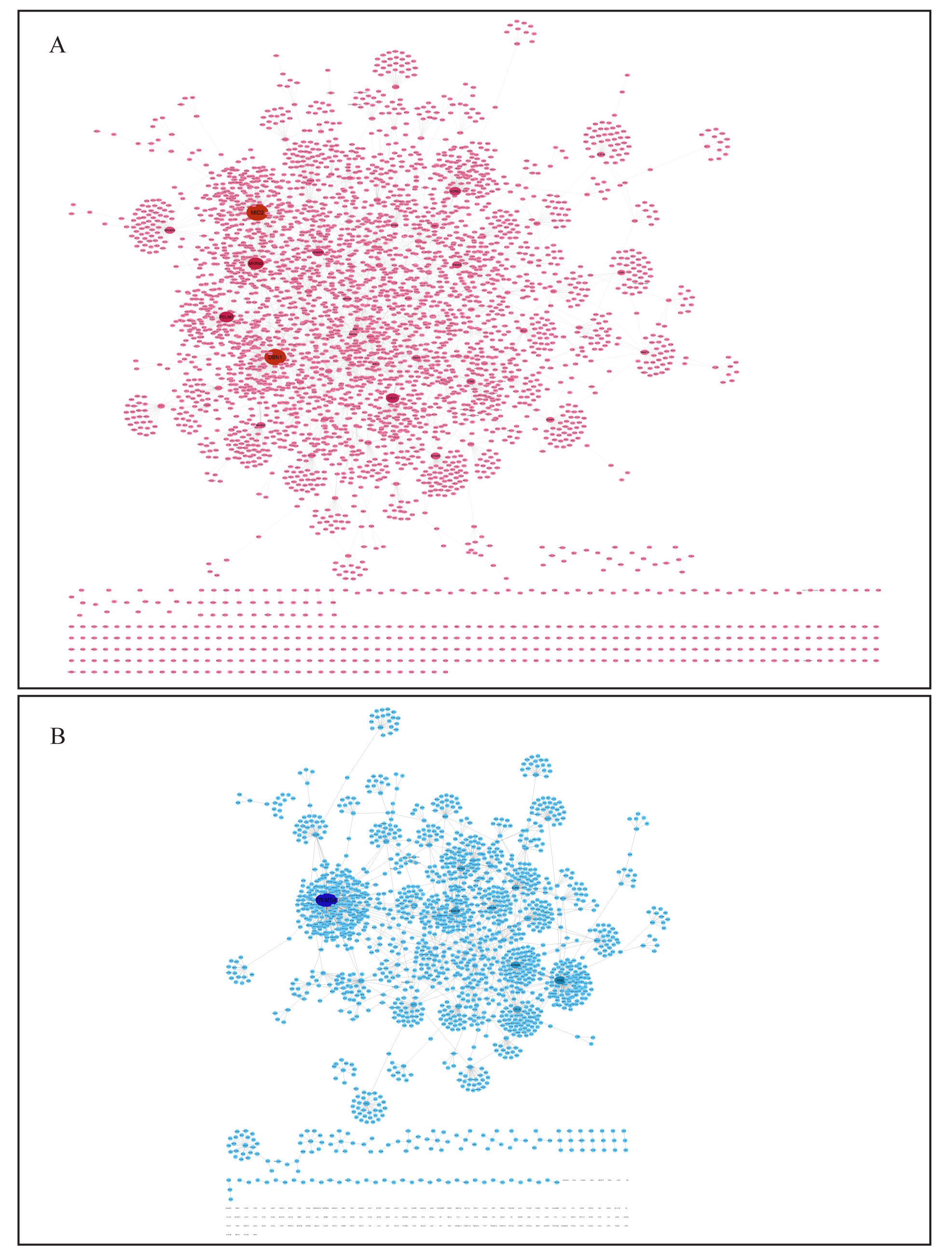

Supplement: S3 Fig — The nodes represent genes and edges represent interaction between genes. The red and blue nodes signify the up-regulated and down-regulated DEGs, respectively. The node size and color indicate the degree value. DEG: Differentially expressed gene; PPI: Protein-protein interaction. (TIF) [file pone.0289535.s003.tif]
